# Synthesis of graphene quantum dots and their applications in drug delivery

**DOI:** 10.1186/s12951-020-00698-z

**Published:** 2020-10-02

**Authors:** Changhong Zhao, Xuebin Song, Ya Liu, Yifeng Fu, Lilei Ye, Nan Wang, Fan Wang, Lu Li, Mohsen Mohammadniaei, Ming Zhang, Qiqing Zhang, Johan Liu

**Affiliations:** 1 School of Life Sciences and Technology, Xinxiang Medical University, Xinxiang, 453003 P. R. China; 2 Electronics Materials and Systems Laboratory, Department of Microtechnology and Nanoscience, Chalmers University of Technology, 412 96 Gothenburg, Sweden; 3 SHT Smart High-Tech AB, 411 33 Gothenburg, Sweden; 4 Department of Health Technology, Technical University of Denmark, 2800 Kongens Lyngby, Denmark; 5 School of Automation and Mechanical Engineering, SMIT Center, Shanghai University, No 20, Chengzhong Road, Box 808, ShanghaiShanghai, 201800 China

**Keywords:** Graphene quantum dots, Top-down, Bottom-up, Drug delivery, Delivery-release mode

## Abstract

This review focuses on the recent advances in the synthesis of graphene quantum dots (GQDs) and their applications in drug delivery. To give a brief understanding about the preparation of GQDs, recent advances in methods of GQDs synthesis are first presented. Afterwards, various drug delivery-release modes of GQDs-based drug delivery systems such as EPR-pH delivery-release mode, ligand-pH delivery-release mode, EPR-Photothermal delivery-Release mode, and Core/Shell-photothermal/magnetic thermal delivery-release mode are reviewed. Finally, the current challenges and the prospective application of GQDs in drug delivery are discussed.
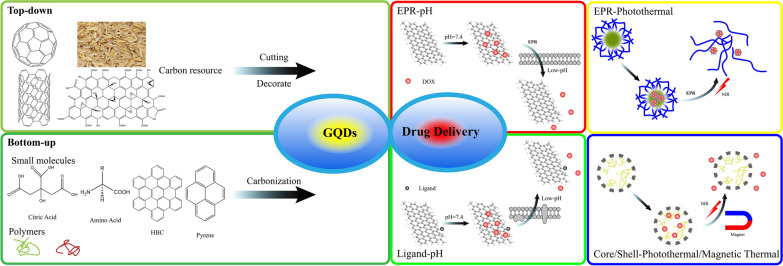

## Introduction

GQDs are graphene blocks with two-dimensional (2D) transverse size (less than 100 nm) [1] and excellent chemical [2], physical [3], and biological properties [4, 5]. An ideal GQD consists of only one atomic layer of carbon atoms. However, most of the synthesized GQDs also contain functional groups like oxygen and hydrogen, and usually have multiple atomic layers with sizes less than 10 nm [6].

Due to its small size, GQD has a better prospect in biomedical applications than graphene or graphene oxide (GO) [7]. However, prior to designing GQDs into practical applications, their biocompatibility and toxicity are still the main concerns. Studies have shown that GQDs have good biocompatibility [8–10] and low biotoxicity [11, 12]. Xie et al. [13] studied the cytotoxicity and autophagy induction of three kinds of GQDs, including cGQDs (HOOC-GQDs), hGQDs (HO-GQDs), aGQDs (H_2_N-GQDs), using lung cancer A549 cells as models. The results showed that hGQDs were the most toxic, leading to significant cell death at 100 μg/mL of hGQD concentration, while cGQDs and aGQDs showed no cytotoxicity within the measured concentration range. Besides cGQDs, aGQDs, and hGQDs can induce autophagy to varying degrees, as shown in Table 1. Further analysis of autophagy pathway showed that all GQDs could significantly activate p-p38MAPK, while p-ERK1/2 was inhibited by aGQDs and hGQDs but activated by cGQDs. The aGQDs and cGQDs inhibited p-JNK and hGQDs activated p-JNK. On the other hand, Akt was activated by hGQDs, but inhibited by aGQDs. The inhibition of 3-MA on autophagy significantly increased the cytotoxicity of GQDs, suggesting that autophagy has a protective effect on the toxicity of GQDs. The results show that cGQDs have better biocompatibility and more potential in biological applications. Generally, the bioavailability of nanoparticles plays an important role in their safety [14]. However, the amount of cellular uptake showed no significant difference between cGQDs and hGQDs, indicating that bioavailability of GQDs may not explain the difference in toxicity of these GQDs. Although the exact reason is still not clear, the autophagy induction abilities of such GQDs could explain the differences of their toxicity profiles. It was found that cGQDs appears to be even inert in autophagy activation, both aGQDs and hGQDs induced cellular autophagy to various degrees except for cGQDs [13]. Interestingly, hydroxylation was thought to be able to enhance the biocompatibility of nanoparticles [15], but when it comes to GQDs, hGQDs showed the highest toxicity of the three GQDs. Therefore, the influence of surface chemistry on the safety of nanomaterials should not be overgeneralized, and the risk assessment of nanomaterials needs to be handled in a case-by-case manner.

**Table 1:** Effects of three GQDs on autophagy-inducing related genes

	p-p38MAPK	p-ERK1/2	p-JNK	Akt
cGQDs	+ +	+	−	
hGQDs	+ +	−	+	+
aGQDs	+ +	−	−	−

“ +  + ” indicates significant activation; “ + ” indicates activation; “−” indicates inhibition

In addition, the monoatomic layer planar conjugate structure, large specific surface area and oxygen-containing functional groups on the surface of GQDs can provide significant active sites and spacious environment to load and carry various drugs/genes/small molecules. Similar with graphene, GQDs have capability to bind with a variety of aromatic bimolecular through the π–π stacking interaction and/or electrostatic binding. However compared with graphene sheets, GQDs exhibit improved biocompatibility and minimal toxicity [16–18], making them more promising materials for delivering of biologically active cargoes into living systems. In recent years, many researchers have been dedicated to the application of GQDs in drug delivery systems [19–21]. Although, study in this field is still in its primary development period, a comprehensive review article focusing on the applications of GQDs in drug delivery is highly demanded.

In this review, we outline the recent advancements in synthesizing of GQDs together with an analysis and comparison study on their pros and cons and suitable applications. Moreover, the applications of GQDs in drug delivery are summarized and discussed, while various GQDs based drug delivery-release modes are reviewed and compared.

## Synthesis methods of GQDs

**Fig. 1:**
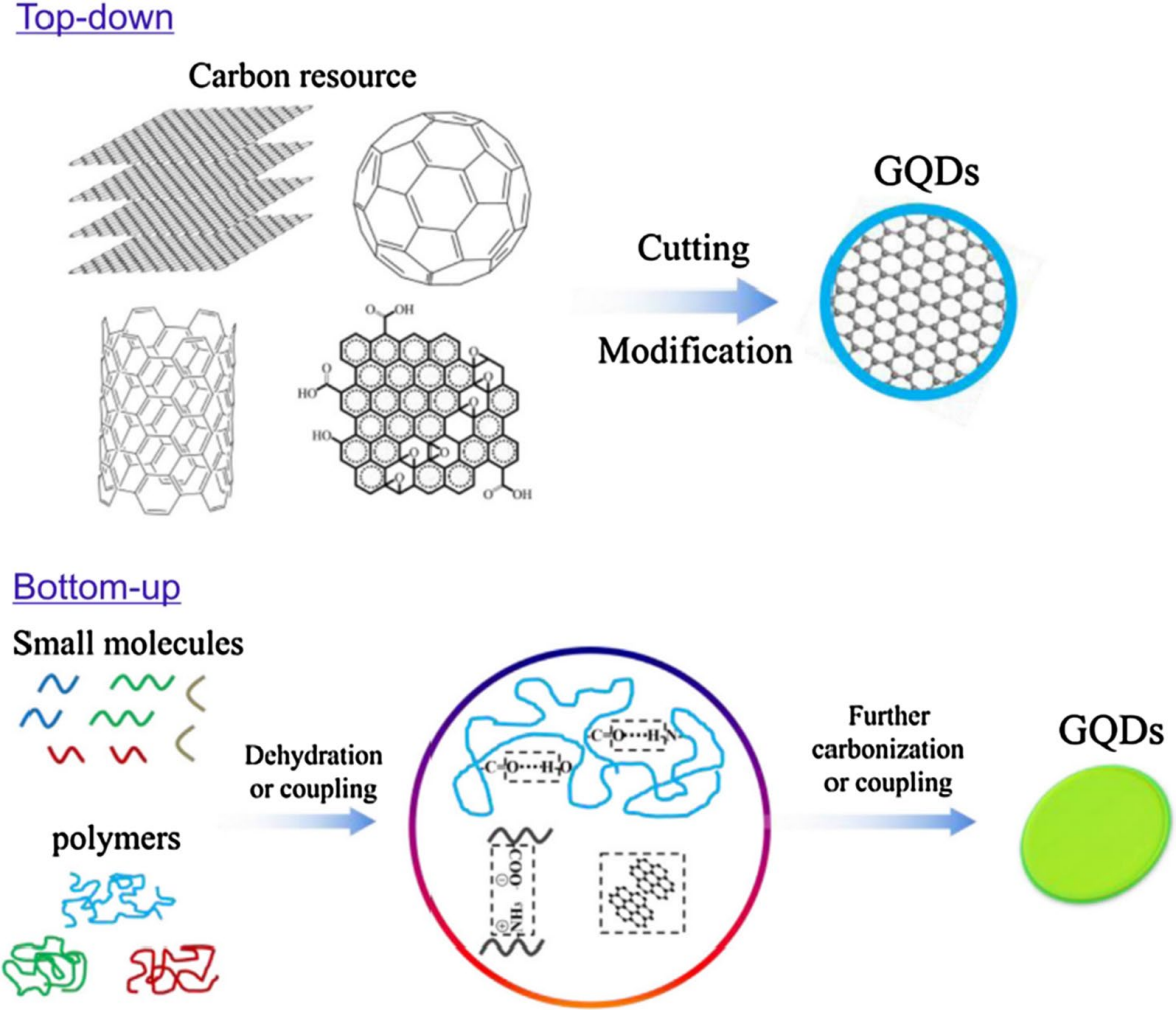
Two main approaches were adopted to prepare fluorescent GQDs: the “top-down” splitting route from different carbon sources and “bottom-up” method from small molecules or polymers (reprinted/reproduced with the permission of Ref. [23], copyright 2017, Nano Today)

The existing methods for GQDs synthesis can be generally divided into top-down and bottom-up processes (Fig. 1) [22]. As the bottom-up methods, synthesis of GQDs requires complex reaction steps and specific organic materials, making it difficult to optimize the conditions. Therefore, it is preferred to use the top-down approach, which is to cut large blocks of carbon materials into small pieces. The raw materials needed for this method are abundant carbon materials, which are cheap and easy to obtain, also the method is relatively straightforward and easy to synthesize GQDs.

### Top-down strategy

There are many methods for GQDs synthesis based on top-down processes, including chemical oxidation method [24–26], hydrothermal method [27, 28], ultrasonic assisted method [29], electrochemical oxidation method [30], chemical vapor deposition (CVD) method [31–33], and pulsed laser ablation (PLA) technique [34–37], or a combination of the above different approaches [38].

#### Chemical oxidation method

Chemical oxidation method, also known as oxidation cutting method, is a very widely used method, in which carbon bonds of graphene, GO or carbon nanotubes are usually destroyed by H_2_SO_4_, HNO_3_ or other oxidants [39–43].

**Fig. 2:**
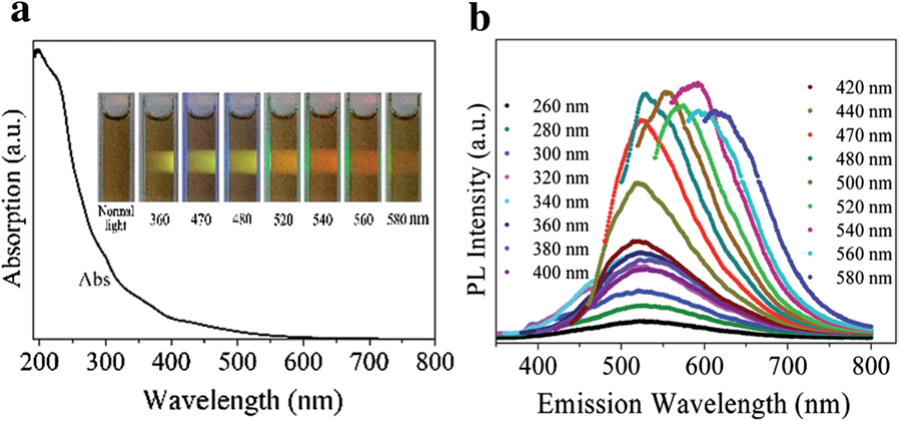
**a** UV–vis absorption spectrum of the GQDs at different excitation wavelengths. Inset: optical photograph of the GQDs dispersed in water under different wavelengths of irradiation. **b** PL spectra of the GQDs at excitation wavelengths from 260 to 580 nm (reprinted/reproduced with the permission of Ref. [44], copyright 2015, RSC Advances)

Liu et al. [44] developed an experimental system, which used Vulcan XC-72 carbon black as carbon source and strong oxidant concentrated nitric acid reflux to prepare high purity GQDs. The yield and purity of GQDs were 75 wt% and 99.96 wt% respectively. At different excitation wavelengths, the prepared GQDs exhibited multicolor photoluminescence (PL) from green to red (Fig. 2).

In order to avoid the use of concentrated acids and the introduction of metal impurities, Lu et al. [45] synthesized GQDs with black carbon as precursor and hydrogen peroxide (H_2_O_2_) as the oxidant in a pot of hydrothermal method without any additional post-treatment steps. The diameter of synthesized GQDs was 3.0–4.5 nm. The whole synthesis process takes only 90 min, and has good light stability, salt tolerance, low toxicity and good biocompatibility. Compared with many other reported methods, it is a more green and faster method for GQDs synthesis. After that, Halder et al. [46] also used GO as the precursor, oxidized and cracked it in 2 h with the help of H_2_O_2_, to obtain GQDs products, which also did not need further purification steps.

Due to the use of strong oxidants such as H_2_SO_4_ and HNO_3_, the chemical oxidation method is not very safe, and the generated chemical waste is liable to pollute the environment.

#### Hydrothermal method

Hydrothermal method is a simple and rapid method for preparing GQDs [47–50]. GQDs can be finally obtained using a variety of macromolecular or small molecular substances [51] as the starting materials through high temperature and pressure [52–55]. The principle is to break the bonds between carbon materials to form GQDs via high temperature under high pressure [56–59].

**Fig. 3:**
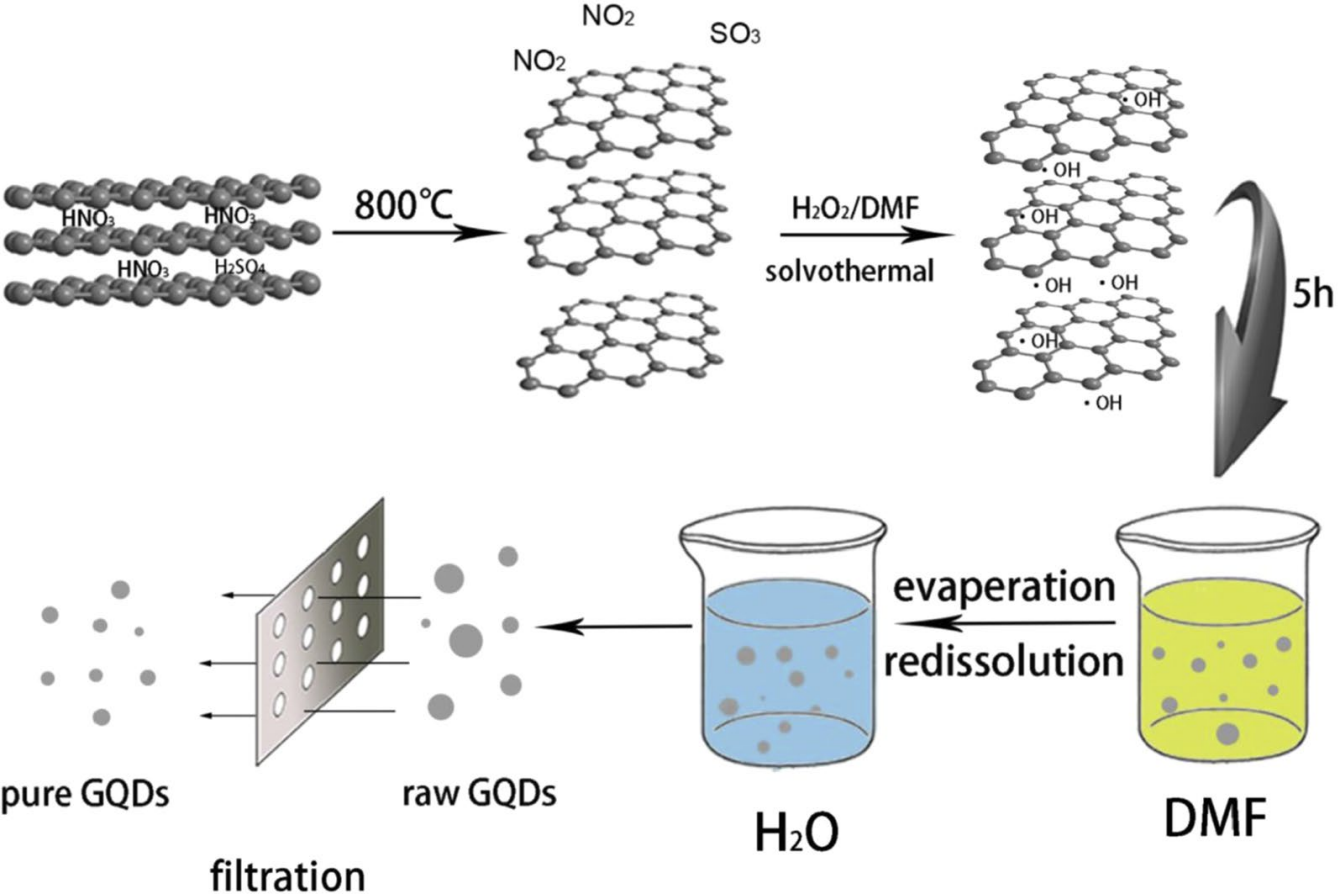
Schematic representation of GQDs prepared by solvothermal method (reprinted/reproduced with the permission of Ref. [60], copyright 2016, Optical Materials)

Tian et al. [60] used H_2_O_2_ to synthesize GQDs in N, *N*-dimethylformamide (DMF) environment by one-step solvothermal method. In the whole preparation process, the use of concentrated sulfuric acid and nitric acid to treat raw materials was completely avoided, whereas no impurities were introduced (Fig. 3). High purity GQDs could be obtained by evaporation/re-dissolution and filtration without dialysis. The results showed that the diameter and thickness of GQDs were mainly distributed within the range of 20–40 nm and 1–1.5 nm, respectively. Under neutral conditions, the quantum yield (QY) was 15%. The PL signal represented a good stability under different pH conditions, which indicates that it has broad application prospects in different environments. This method has many advantages such as low cost, high quantum yield, no requirement for dialysis and purification, simple experimental setup, etc. The prepared GQDs were environmentally friendly and displayed sound water solubility to represent their promising applications in the field of biomedical and bioelectronic devices.

Recently, Zhang et al. [61] successfully synthesized reduced graphene oxide quantum dots (rGOQDs) in 5 h. They used graphite as the starting material to prepare GO by an improved Hummers’ method, and then GO and DMF were utilized as the raw materials for further hydrothermal treatment in a poly(tetrafluoroethylene) (Teflon)-lined autoclave at 200 °C. The QY of the synthesized rGOQDs was 24.62%, and the surface doping was nitrogen (N) extracted from DMF. They also studied zebrafish by rGOQDs, which provided valuable reference for the biocompatibility of bio-probes in vivo.

In order to make full use of crop biomass, some researchers [62] used rice husk as the raw material to produce high-quality GQDs by hydrothermal method. The mass fraction of QY is about 15 wt%. The prepared GQDs showed good colloidal stability in water with bright and adjustable PL signals. Experiments suggested that, the synthesized GQDs has good biocompatibility and can be easily translocated into cytoplasm, to be used for cell imaging. In addition, mesoporous silica nanoparticles (MSNs) as the by-products were synthesized during the synthesis of GQDs.

Hydrothermal method can be used to dope many elements or groups, and the raw materials come from a wide range of composites [63]. Moreover, the hydrothermal method can be combined with chemical oxidation method to prepare different GQDs [64–66]. However, it suffers from the safety issue, because of the high temperature and pressure, and also it generally takes a long time, usually at least 5 h [67, 68].

#### Ultrasound assisted method

**Fig. 4:**
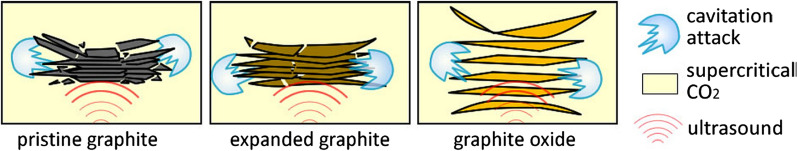
Illustration of the exfoliation process of pristine graphite, expanded graphite and graphite oxide in ultrasonic-assisted scCO_2_ process (reprinted/reproduced with the permission of Ref. [71], copyright 2017, Ultrasonics Sonochemistry)

Ultrasonic technology is also a common method for material synthesis [69, 70]. Under the action of ultrasound, tens of thousands of small bubbles will be formed in the liquid, and the mechanical force generated can destroy the carbon–carbon bonds, thus cutting into GQDs (Fig. 4).

Gao et al. [71] prepared three kinds of GQDs of pristine graphene quantum dots (PGQDs), expanded graphene quantum dots (EGQDs) and graphene oxide quantum dots (GOQDs) using natural graphite, expanded graphite, and oxide graphite as the raw materials in a supercritical CO_2_/H_2_O system assisted by ultrasound. The experimental results show that this method is an environmentally friendly, low-cost, fast and large-scale synthesis method of GQDs, which it can provide an alternative green route for the production of various GQDs, especially PGQDs.

Balaji et al. [72] calcined the latex of *Calotropis gigantea* to 300 °C and extracted it with ethanol. The pure rGOQDs can be obtained by re-dispersing the extract in Milli-Q water with a 15 min sonicate treatment and further centrifuging at 5000 rpm. The particle size of rGOQDs ranged from 2–8 nm and showed green fluorescence in the long ultraviolet range of 360–520 nm. It can be used to design more environmentally friendly and economical Pb^2+^ fluorescent probes, since, it provides a simple and suitable method for the selective and sensitive detection of Pb^2+^ in water purification process. In addition, the rGOQDs were also prepared for free radical scavenging and bioimaging applications. They showed the advantages of stability, cost-effectiveness, good biocompatibility and environmental protection to play an important role in the field of nanotechnology-based biomedicine in the near future. Although, combination of hydrothermal and ultrasound-assisted methods have shown to improve their drawbacks in the fabrication of GQDs [73, 74].

#### Electrochemical oxidation method

In the electrochemical oxidation method process, carbon–carbon bonds of graphite, graphene, or carbon nanotubes are oxidized and decomposed into GQDs at a high redox voltage (+ 1.5 to + 3 V) [30, 75].

**Fig. 5:**
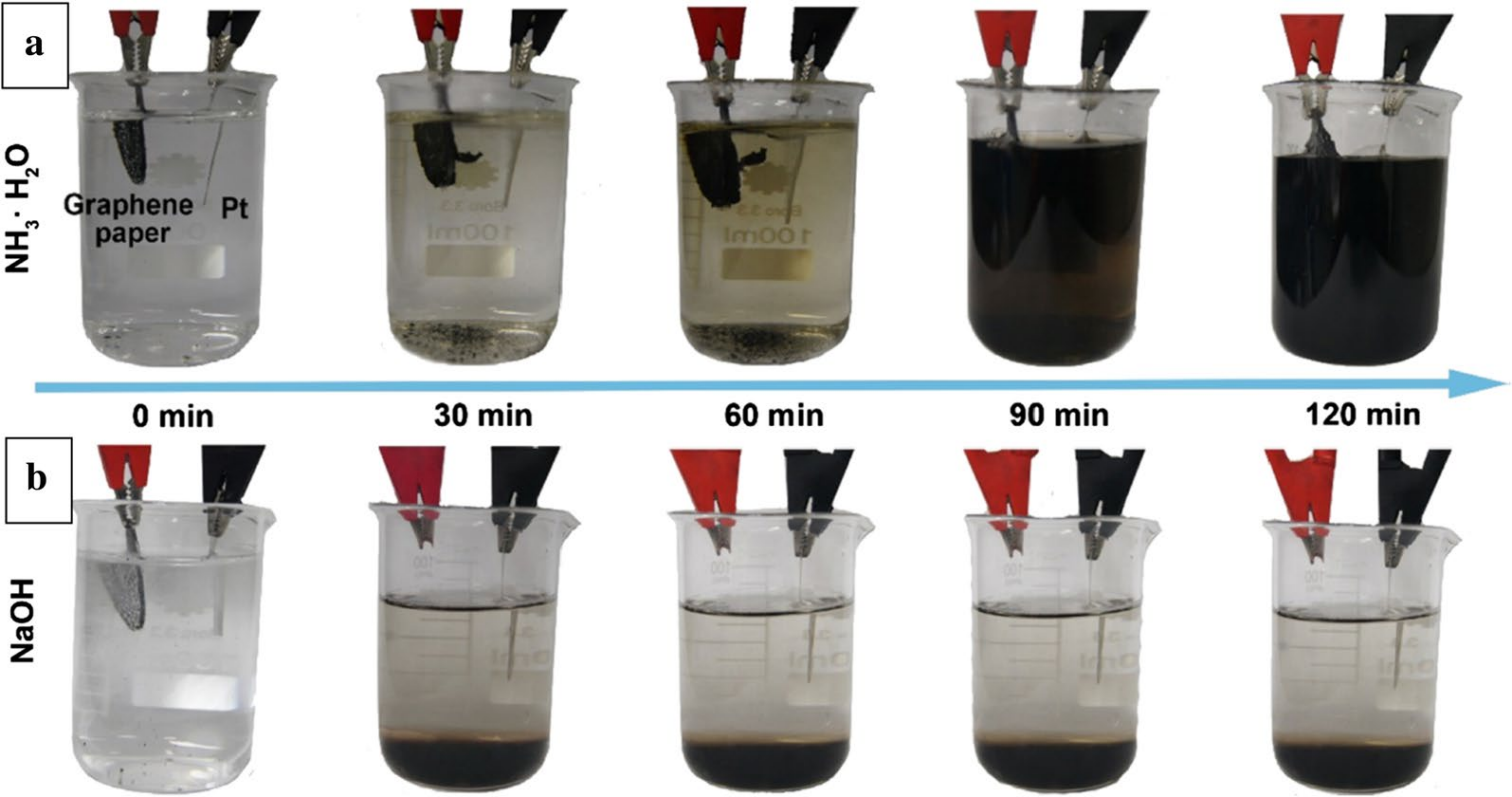
The electrolytic process of graphene paper in **a** ammonia and **b** NaOH solutions under different reaction times (reprinted/reproduced with the permission of Ref. [76], copyright 2018, Langmuir)

In order to controllably and efficiently prepare highly crystalline GQDs in aqueous systems, researchers [76] have developed a weak electrolyte (such as ammonia solution) electrochemical method to enhance the oxidation and cutting process, thereby achieving high yield of GQDs. The af-GQDs were prepared using a circular graphene paper as the anode, a Pt sheet as the cathode, and an ammonia solution (nitrogen source) as the electrolyte and operated in a constant voltage mode (30 V) for 2 h in an electrochemical cell (Fig. 5). GQDs had a size of 3–8 nm and QY of 28%, which was approximately 28 times greater than that of the strong electrolytes (such as borax solution). At the same time, GQDs also showed significantly better crystallinity than the bottom-up GQDs. In addition, the amino functionalization of GQDs can be controlled by controlling the electrolyte concentration. In addition, this method can also be used in other weak electrolytes (such as HF and H_2_S) and anode precursors (such as graphene/graphite paper, carbon fiber and carbon nanotubes) to prepare other types of GQDs.

In a study conducted by by Chen et al. boron-doped graphene quantum dots (BGQDs) were synthesized by potentiostatic electrolysis [75]. Firstly, they put high purity graphite rod (carbon source) as the anode and PT sheet as the cathode into borax solution (boron source) of pH ≈ 7. Oxidization and decomposition of graphite at high redox voltage (3 V) for 2 h led to the production of BGQDs. Then, a 0.22 μm microporous nylon membrane filtration and dialysis bag (retained molecular weight of 3500 Da) were used to obtain a high-purity BGQDs solution.

The GQDs solution prepared by the electrochemical oxidation method has high stability, but the pretreatment of raw materials and the purification of GQDs products take a long time, and the QY is relatively low, which makes it difficult to achieve large-scale production of GQDs.

#### Other methods

Due to the unique structure and excellent properties of GQDs, researchers have reported more and more preparation methods based on the popular techniques, such as chemical vapor deposition (CVD) [33, 77] and pulsed laser ablation (PLA) [78, 79].

**Fig. 6:**
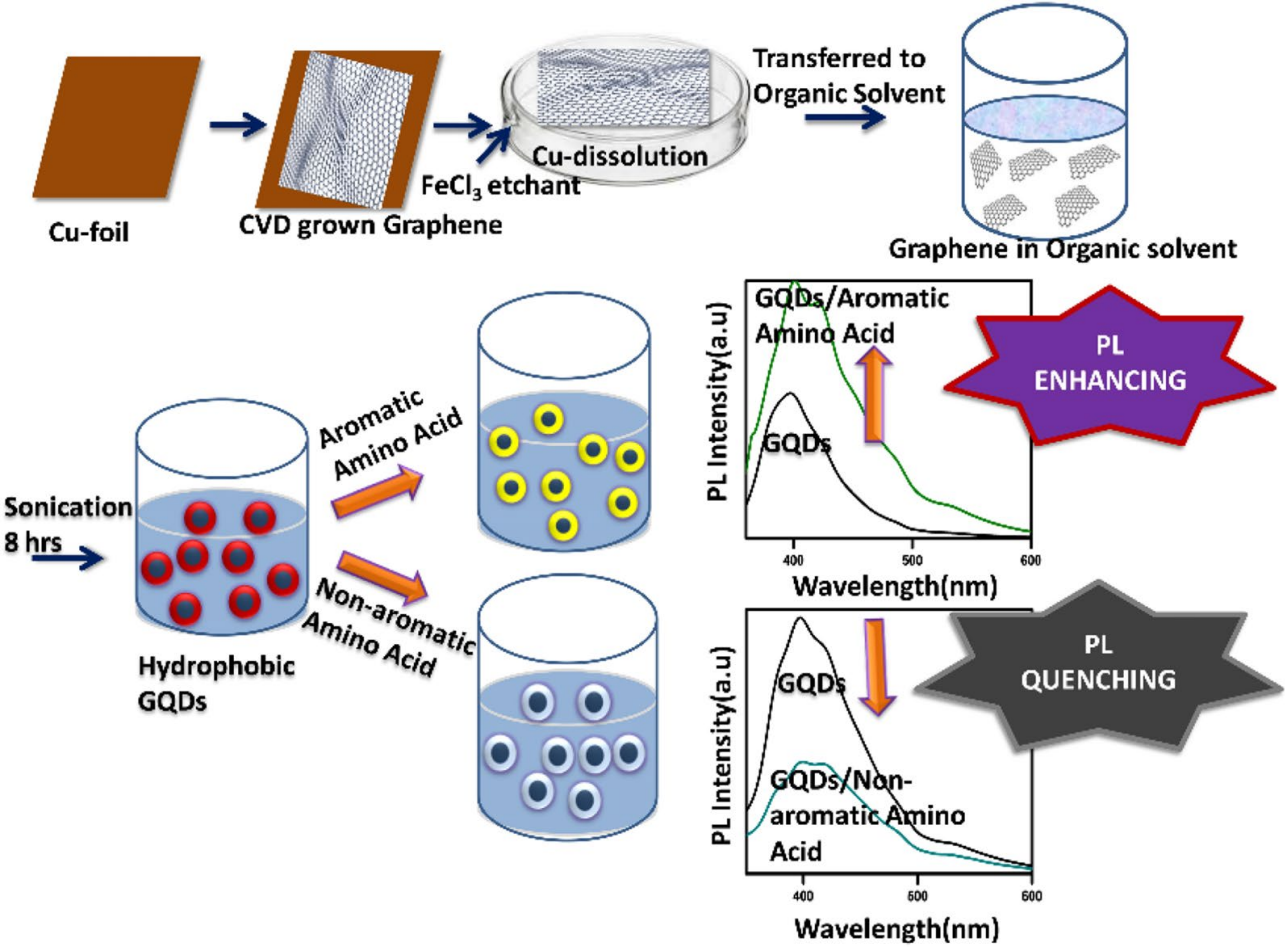
Schematic representation of the strategy employed to synthesize h-GQDs and fabrication it as a sensing system which can distinguish between aromatic and non-aromatic amino acids (reprinted/reproduced with the permission of Ref. [31], copyright 2017, ChemistrySelect)

For example, Deka et al. [31] prepared a PL sensor based on hydrophobic graphene quantum dots (h-GQDs) using acetylene and hydrogen as raw materials by CVD, which can distinguish aromatic and non-aromatic amino acids (Fig. 6). They first grew graphene on the Cu substrate through a custom CVD system, then transferred it directly to *n*-hexane which was followed by obtaining h-GQDs after 8 h of ultrasonic treatment. This is the first report on the direct synthesis of CVD-assisted h-GQDs, which can form highly stable dispersions in organic solvents without functionalization, doping or binding with other molecules.

**Fig. 7:**
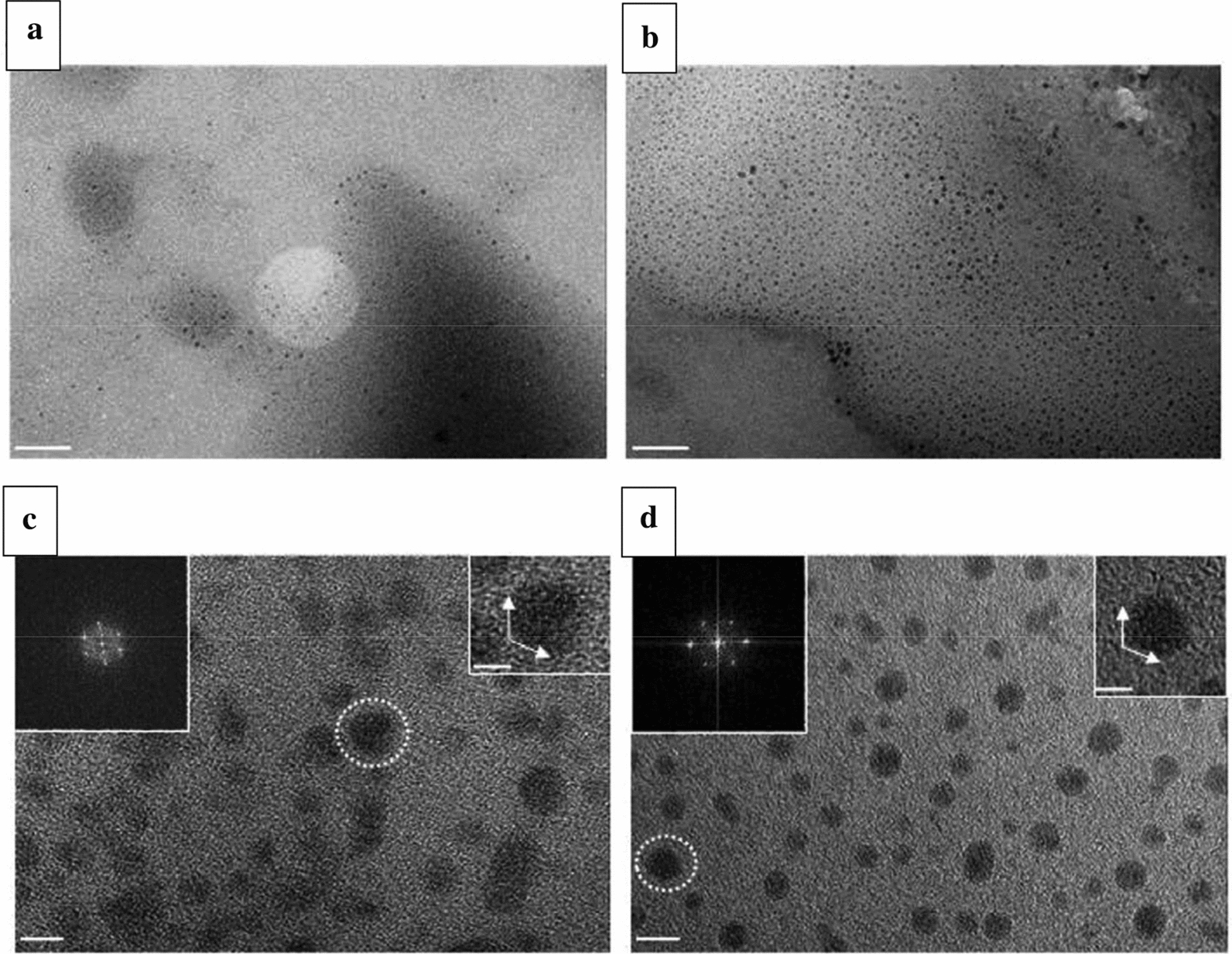
HR-TEM images of e-GOQDs and h-GQDs. **a** TEM image of e-GOQDs and **b** h-GQDs. They both showing the uniform round shape and size distribution of 1 ~ 5 nm. Scale bar 50 nm. **c** HR TEM image of e-GOQDs and **d** h-GQDs. Insets are the 2D FFT patterns (left). They both show high quality crystalline hexagonal patterns of these quantum dots. Scale Bar 5 nm. Right side insets show the edge structure of e-GOQDs and h-GQDs. Scale bar 2 nm (reprinted/reproduced with the permission of Ref. [34], copyright 2016, Scientific Reports)

In order to open up a new way to prepare GQDs, Kang et al. [34] prepared GQDs from multi-walled carbon nanotubes (MWCNTs) by PLA. They first dispersed MWCNTs as carbon precursors in n-hexane and ethanol, respectively. Ultrasound treatment was then carried out in the solution for 2 h to achieve uniform dispersion of MWCNT (Fig. 7). GQDs were fabricated rapidly by transferring 50 mL solution to glass bottle and then using a 6 min pulsed laser peeling (PLE) process on a fixed bottle. The synthesized GQDs represented an obvious blue PL with the QY of 12%, and showed sufficient brightness and resolution, to make them suitable for photoelectric applications.

In addition, some researchers [80–84] have prepared GQDs by other methods, but it has not been widely used due to their disadvantages such as difficult particle size control, low yield, long reaction time and complex process.

### Bottom-up strategy

The bottom-up approach generally includes microwave method [73, 85–87], molecular carbonization [88–90], and electron beam irradiation (EBI) methods [91, 92]. Generally, small molecules [93, 94] such as citric acid (CA) [95–97], amino acid [98, 99], phenyl compounds [91, 100–103], or small molecule sugar [104–106] are used as the starting material.

#### Microwave method

The long reaction time of hydrothermal method is a common problem, so microwave technology has become a fast heating method, which is widely used in the preparation of nanomaterials [107–109]. It not only shortened the reaction time, but also increased the yield.

**Fig. 8:**
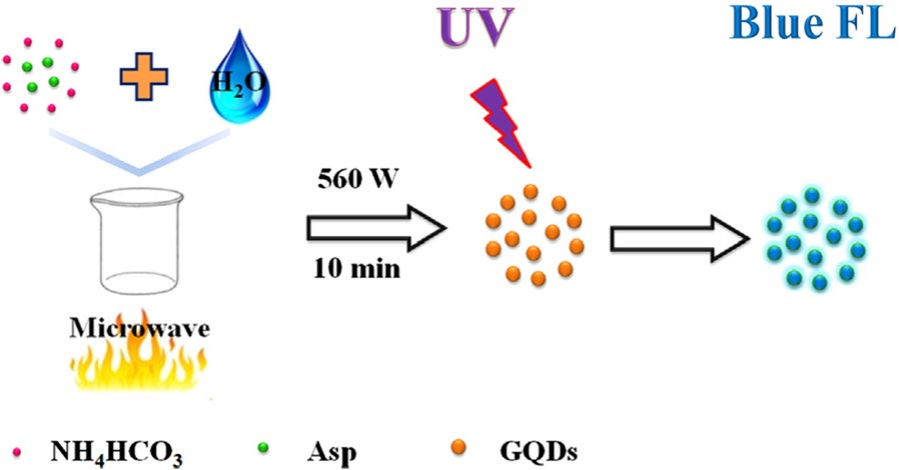
Schematic illustration of the preparation process for the GQDs (reprinted/reproduced with the permission of Ref. [110], copyright 2016, Talanta)

Zhang et al. [110] used aspartic acid (Asp) and NH_4_HCO_3_ as raw materials, DI water as solvent, purified GQDs by microwave irradiation for 10 min and dialysis membrane for 7 h (Fig. 8). The prepared GQDs showed strong blue fluorescence and QY of 14%. The strong fluorescence quenching effect of Fe^3+^ on GQDs can be used for highly selective detection in general metal ions. GQDs is also sensitive to pH value (2–12), which shows that it has great potential for optical pH sensors. In addition, GQDs can be directly used as fluorescent probe for cell imaging due to its low cytotoxicity and high photostability.

In order to obtain GQDs with good biocompatibility, sensing and in vivo bioimaging capabilities, Campbell et al. [111] used glucosamine-HCl solution as a carbon source and added different dopant precursors (sulfur thiourea or benezeneboronic acid) to synthesize various GQDs, including N-GQDs, NS-GQDs, and BN-GQDs. After the mixed solution was subjected to microwave treatment for 40 min, it was treated with dialysis membranes for 7 days, and GQDs with QY of 15–20% were obtained. After examining the cytotoxicity and pH fluorescence response of the prepared GQDs, it was found that they have a great potential in drug delivery, pH-sensing of cancerous environments, and multicolor visible/near-infrared (NIR) fluorescence imaging.

The microwave method greatly shortens the time for synthesizing GQDs, which can be obtained in a few minutes, and can be doped with various elements, which enriches the types of GQDs and expands the functions of GQDs.

#### Molecular carbonization method

The preparation of GQDs by molecular carbonization is an environmentally friendly and simple method [90, 112]. Its principle is to use suitable organic molecules or polymers for dehydration and further carbonization [113–115].

**Fig. 9:**
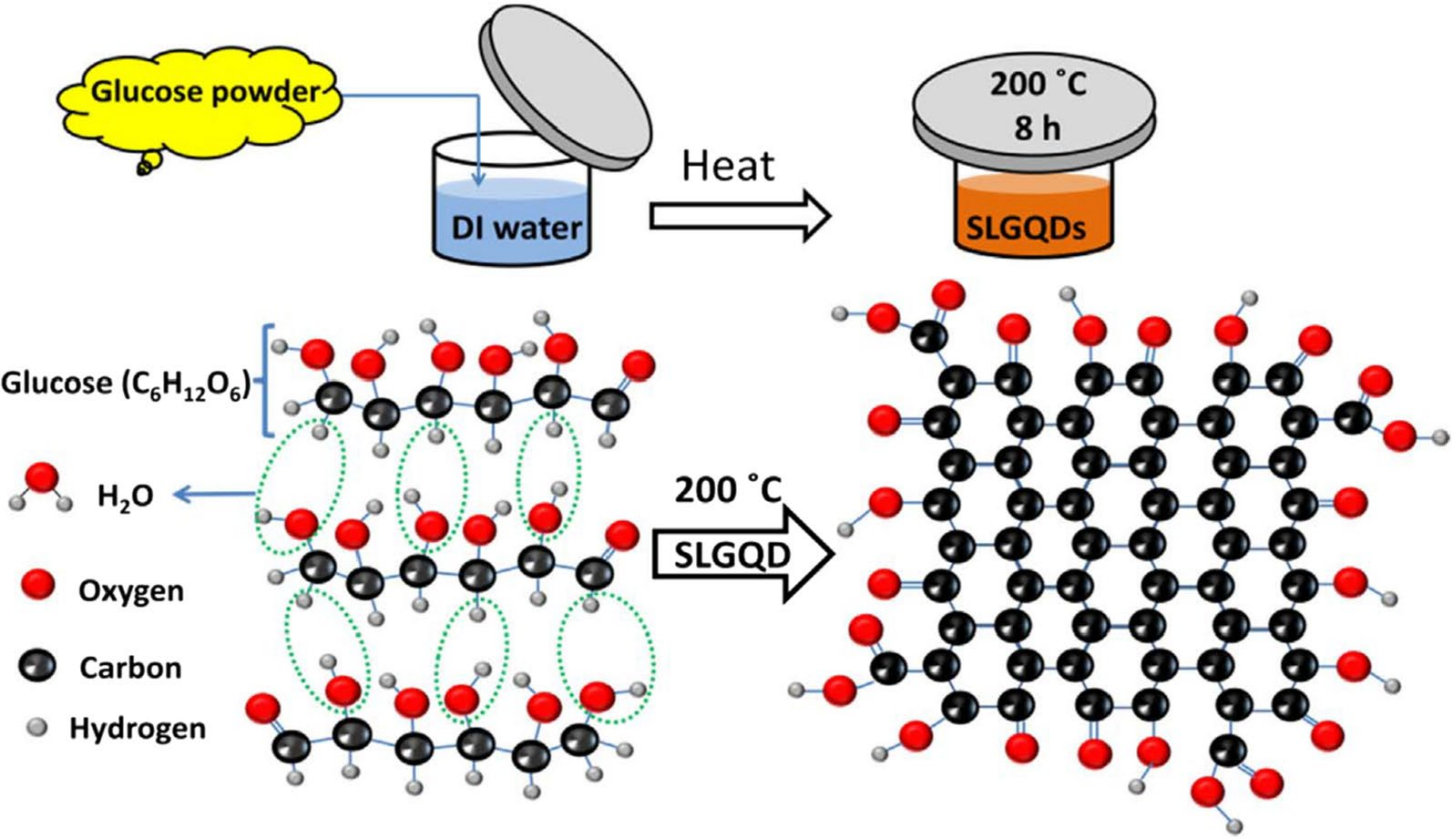
Formation mechanism of the SLGQDs via a hydrothermal method at 200 °C for 8 h (reprinted/reproduced with the permission of Ref. [116], copyright 2017, Journal of Luminescence)

Bayat et al. [116] synthesized low cost and high yield green photoluminescent single-layer graphene quantum dots (SLGQDs) using DI water as solvent and glucose as precursor. The synthesized SLGQDs were uniformly dispersed without obvious aggregation, and their average size was about 8 nm. The maximum emission wavelength was about 540 nm. The formation mechanism of SLGQDs was as follows (Fig. 9). First, dehydration of glucose molecule to form C=C and basic unit of graphene structure through hydrothermal reaction; second, interaction of hydrogen atoms of glucose molecule with hydroxyl group of adjacent glucose molecule to form water molecule; finally, covalent interaction of carbon atom to form GQDs. The prepared SLGQDs have the advantages of low cost, high yield and large scale.

In another report, Teymourinia et al. [117] prepared GQDs with corn flour as green precursor. The diameter of synthesized GQDs was 20–30 nm. Broad emission centered at 450 nm was observed in the emission spectrum (PL) of 360 nm excitation wavelength. The excitation spectrum (PLE) showed a broad peak centered at 365 nm.

The application of molecular carbonization method is relatively challenging. Since the size and structure of GQDs cannot be accurately controlled, the GQDs obtained using this method is multi-dispersive [118–120].

#### Electron beam irradiation (EBI) method

**Fig. 10:**
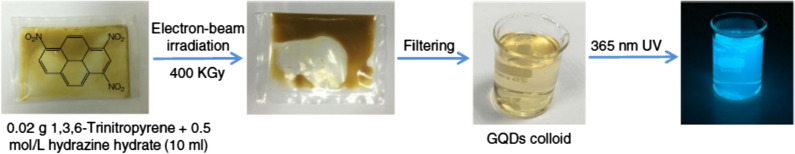
Preparation procedure of GQDs by EBI (reprinted/reproduced with the permission of Ref. [122], copyright 2017, Chemical Engineering Journal)

It is worth to note that, the EBI method [121] requires expensive professional equipment and has the risk of being injured by radiation, so it has not been widely used. Wang et al. [122] synthesized single crystal fluorescent GQDs by EBI at room temperature. 1,3,6-trinitropyrene was dissolved in a solution of hydrazine hydrate. Then, it was sealed in a plastic bag after stirring, and irradiated under the titanium window of a dynamitron electron accelerator (Fig. 10). After irradiation, the sample was dialyzed through a 0.22 mm microporous membrane filter and a dialysis bag for 2 d, and finally a GQDs with 32% QY was obtained. Other small molecules such as 1-Nitropyrene, urea, and CA can also be used as precursors to synthesize GQDs at the same conditions.

Generally, GQDs are prepared by cutting carbon materials, including graphene, fullerene and carbon nanotubes, through top-down strategies, including chemical oxidation method, hydrothermal method, ultrasound Assisted method, electrochemical oxidation method, CVD method, and PLA method, or using appropriate organic molecules or polymers as raw materials. Bottom-up strategies include microwave method, molecular carbonization method, and EBI method. Generally, small molecules such as CA, amino acids, phenyl compounds or small molecular sugars are used as starting materials, or different elements are doped to synthesize GQDs with multiple functions. Table 2 summarizes the advantages and disadvantages of different methods.

**Table 2:** Advantages and disadvantages of different methods

	Methods	Advantages	Disadvantages
Top-down strategy	Chemical oxidation Method	It is widely used method at present; it is simple and effective and can be used in large-scale production	It usually needs to use H_2_SO_4_, HNO_3_ or other oxidants, which may cause corrosion or explosions
	Hydrothermal/solvothermal method	It is a green, simple and fast method	Reaction time is long; some raw materials need to be treated by strong oxidant before reaction occurs; reaction also involves high temperature and high pressure, which may cause combustion or explosion
	Ultrasound assisted method	It can shorten the reaction time and improve the yield	It is difficult to synthesize on a large scale in industry
	Electrochemical Oxidation Method	The GQDs produced are stable and uniform in size distribution	The pretreatment of raw materials and the yield of products is low, so it is difficult to carry out large-scale production
	Other method		It is difficult particle size control, low yield, long reaction time and complex process
Bottom-up strategy	Microwave method	It greatly shortens the reaction time and is green	It is difficult to carry out large-scale production and requires filtration and purification
	Molecular carbonization method	It is an environmentally friendly and simple method	It is impossible to control the size and structure of GQDs accurately; the obtained GQDs are multi-dispersive
	Electron beam irradiation method	It is simple, fast and high yield	It requires expensive professional equipment and has the risk of being injured by radiation, so it has not been widely used

In all GQDs preparation methods, hydrothermal methods are often combined with chemical oxidation methods to break down large molecules such as graphite, fullerene, C_60_, carbon nanotubes, and even crop biomass. The sources of raw materials are very rich, so they are widely used. The microwave method has the advantages of short reaction time, simple operation, cheap equipment, and no pretreatment of raw materials, so researchers can quickly prepare GQDs for further experiments.

## Applications of GQDs in drug delivery

In recent years, the applications of GQDs in drug delivery [123–127], sensors [128–134], bio-imaging [10, 63, 135–140], magnetic hyperthermia [141–143], photothermal therapy [144–148], antibacterial [145, 149, 150], catalyst [69, 151–155], environmental protection [38, 156, 157], and energy [158–163] has made remarkable accomplishment. In order to better apply GQDs to drug delivery, some researchers had used density functional theory (DFT) calculations [164–167], molecular dynamics (MD) simulations [168, 169], or other methods [170, 171] to theoretically study the properties of GQDs. For example, Vatanparast et al. [166] studied the interaction of 5-fluorouracil (FU) with undoped/doped GQDs by DFT calculations. The results showed that AlN and AlP doped GQDs could serve as potential carriers for FU drugs in the nanomedicine domain. Later, they [167] used DFT calculations to study the applications of GQDs and doped GQDs as potential carriers of isoniazid (Iso). The results confirmed that the AlN- and AlP-doped GQDs could be used as potential carriers for drug delivery applications. Recently, they [169] also has studied the effects of different N-functionalities groups in the drug delivery performance of N-GQDs via DFT calculations and MD simulations. The drug release performance of the center N-GQDs is considered to be superior to that of pristine GQDs and edge N-GQDs. This review focuses on the research accomplishment of GQDs in drug delivery.

There are many ways of drug delivery, but simply focusing on drug delivery and ignoring drug release cannot improve the therapeutic effect of drugs. Therefore, more and more researchers have being paying attention to the close relationship between drug delivery and drug release and try to develop a variety of drug delivery-release mode, in order to improve the therapeutic effect of drugs by improving drug delivery and release efficiency.

GQDs is a novel and efficient nano-material for biological therapy. Graphene or graphene-based nanomaterials [145, 172] have been reported for drug delivery and release in order to improve delivery efficiency and enhance therapeutic effects [143, 171–174]. Compared with graphene, GQDs have better water solubility, lower cytotoxicity [61, 150, 175, 176], and larger specific surface area, which makes them more effective drug molecular loading cores [12, 177, 178].

As a member of graphene family and carbon-based nanomaterials, GQDs have a number of advantages over other nanoparticles in the application of drug delivery due to their low toxicity, large surface/volume ratio and their massive capabilities of surface functionalization. When compared to other traditional nanoplatforms, such as polyethylene glycol (PEG), GQDs may provide more bonding sites for chemotherapeutic conjugation and improved cell uptake ability [179]. Compared with quantum dots (QDs) of similar size, GQDs exhibit superior properties such as quantum confinement effects, ability for simultaneous tracking due to their tunable photoluminescence (PL) but relatively lower toxicity due to the lack of heavy metal components [180]. Therefore, GQDs have great potential for biomedical application. Moreover, owing to their planar structure, GQDs possess a large surface area to volume ratio, which allows for higher efficiency of drug loading and delivery [142]. Furthermore, the unique π-orbitals in the sp2-hybridized GQD lattice can be used to bond drugs containing an aromatic ring structure through π–π stacking, without covalent conjugation, which widens the application of GQDs for drug delivery.

**Fig. 11:**
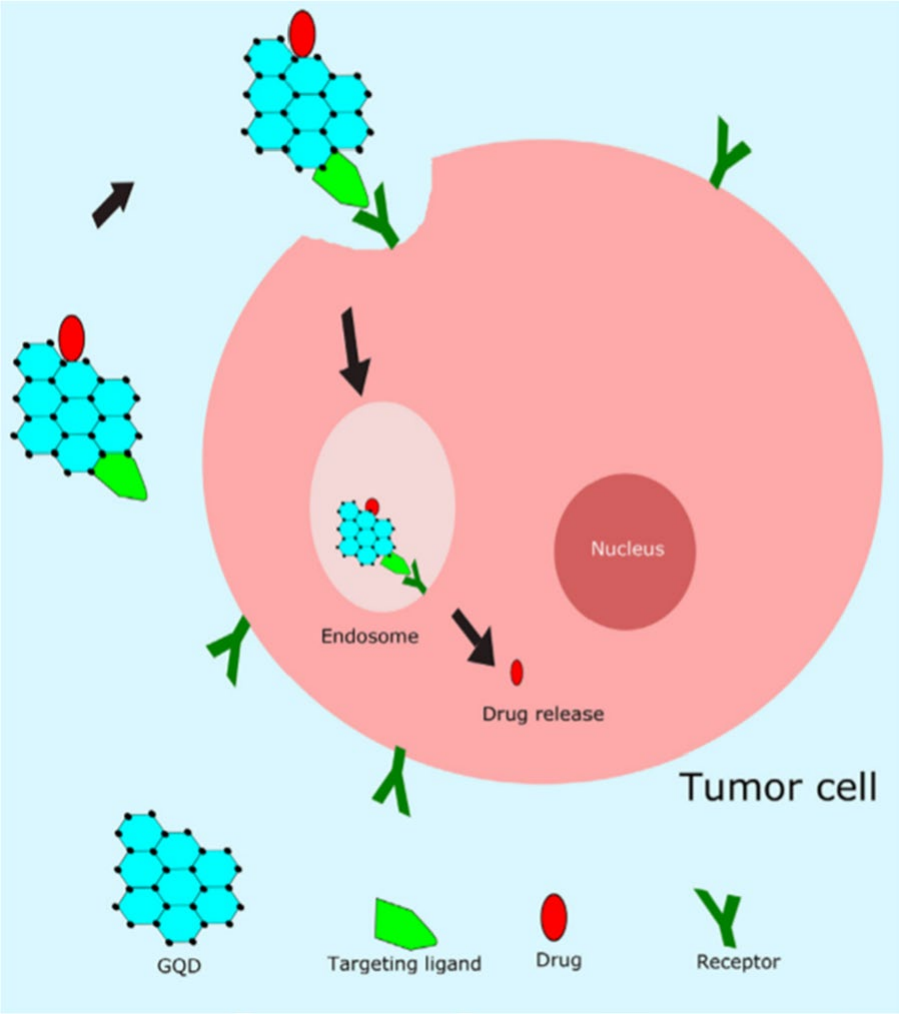
Illustration of receptor-mediated endocytosis of targeting ligand-conjugated GQDs loaded with an anticancer drug into a tumor cell and drug release inside the cell (reprinted/reproduced with the permission of Ref. [181], (reprinted/reproduced with the permission of Ref. [181], copyright 2020, Materials Science & Engineering: C)

A schematic diagram showing the drug delivery and release in GQDs based system is shown in Fig. 11 [181]. First, the drugs are delivered to the target cells by EPR effect or the targeting ligand, then up taken by cells. Similar with other non-degradable nanoparticle drug carriers, drugs released from GQDs via a diffusion process. For example, the adsorbed drugs on GQDs can be released into cytoplasm by desorption and diffusion.

GQDs were proved to be able to across the blood–brain barrier (BBB) and prevent α-synucleinopathy in Parkinson’s disease without surface functionalization [182]. Generally, size and charge of the nanoparticles have important influence on their BBB permeation. Smaller nanoparticles are able to cross the BBB more easily and to diffuse better through the brain. For example, gold nanoparticles under 15 nm were found to be able to cross the BBB without any functionalization. However, gold nanoparticles bigger than 50 nm were failed to across BBB and not found in the brain [183]. Similarly, the small size is considered to the main reason why GQDs can across BBB, and GQDs across biological barriers probably through the transmembrane or the paracellular pathway.

The integration of cancer diagnosis and treatment has been always a concern for the biomedical researchers. Although considerable progress has been made in targeting drug delivery systems to deliver anticancer drugs to specific sites of interest, new nanomaterials are often developed and explored for better drug delivery efficiency. While developing new nanomaterials, new drug delivery-release modes have also been explored. Generally, there are enhanced permeability and retention (EPR)-pH delivery-release mode [184], ligand-pH delivery-release mode [99, 185, 186], EPR-Photothermal Delivery-Release Mode [123], and Core/Shell-photothermal/magnetic thermal delivery-release mode [187, 188]. In addition, other delivery-release modes are generally used to treat non-tumor diseases.

### EPR-pH delivery-release mode

Drug-Loaded Delivery-Release System (DDRS) can be used to deliver drugs to tumor sites through the EPR effect and released in low pH microenvironments for anti-tumor chemotherapy.

**Fig. 12:**
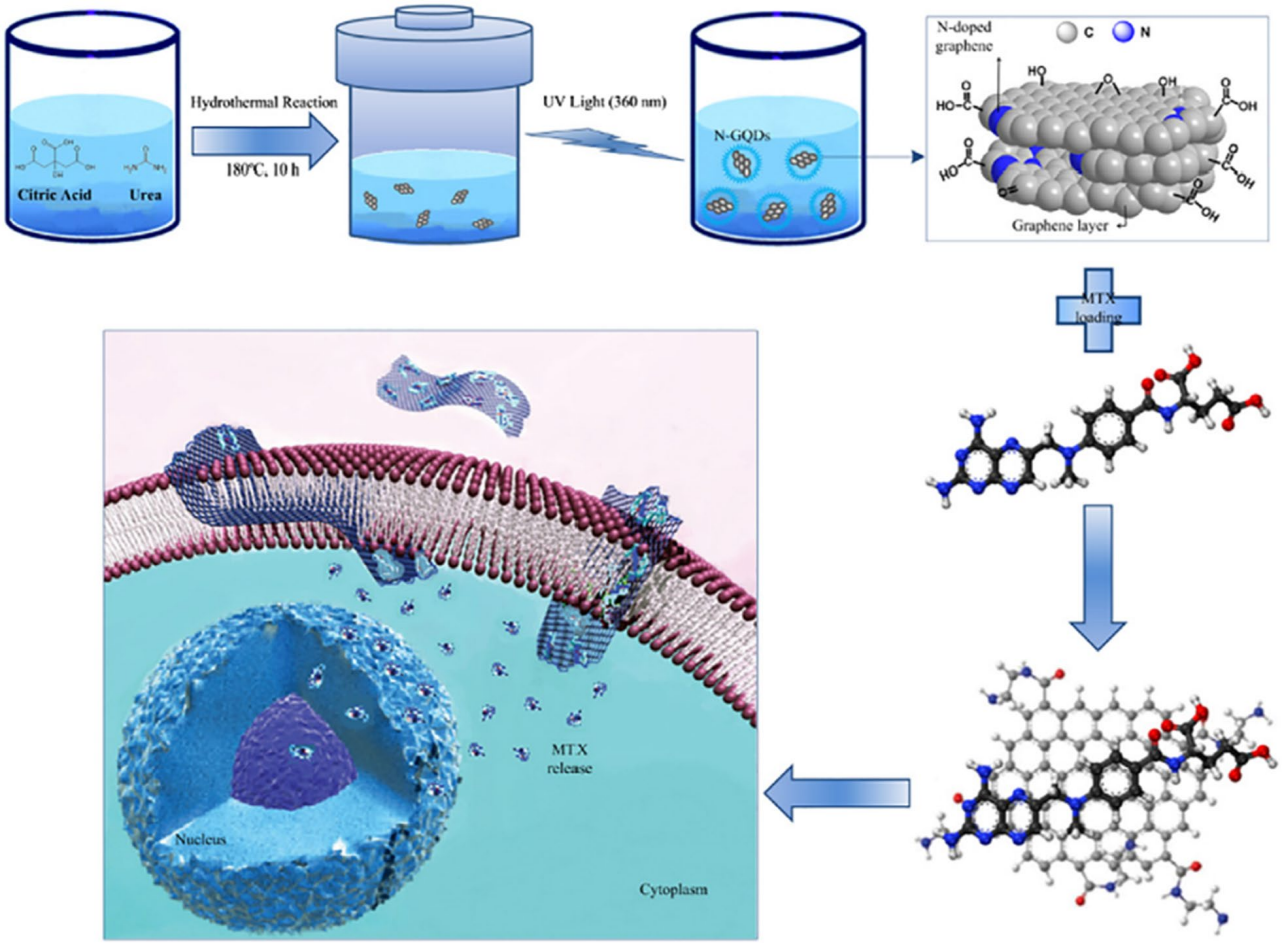
Preparation of N-GQDs and subsequent release of MTX from the surface of N-GQDs in a tumor cell environment (reprinted/reproduced with the permission of Ref. [184], (reprinted/reproduced with the permission of Ref. [184], copyright 2017, Materials Science and Engineering: C)

In a report by Khodadadei et al. [184], blue fluorescent nitrogen-doped graphene quantum dots (N-GQDs) were synthesized by hydrothermal method using CA as the carbon source and urea as the nitrogen source. N-GQDs were loaded with methotrexate (MTX) through π–π superposition interactions, thereby preparing MTX-(N-GQDs) DDRS (Fig. 12). The release of MTX in vivo was simulated by the release of MTX-(N-GQDs) in phosphate buffered saline (PBS) at pH 7.4. In vitro cytotoxicity tests on human breast cancer cells (MCF-7) showed that N-GQDs were well compatible with cells, while MTX-(N-GQDs) had higher cytotoxicity and longer culture time than MTX alone Inside. This study confirms the progress of GQDs as nanocarriers in prolonging the cytotoxicity of drug-loaded cells, thereby better killing cancer cells.

**Fig. 13:**
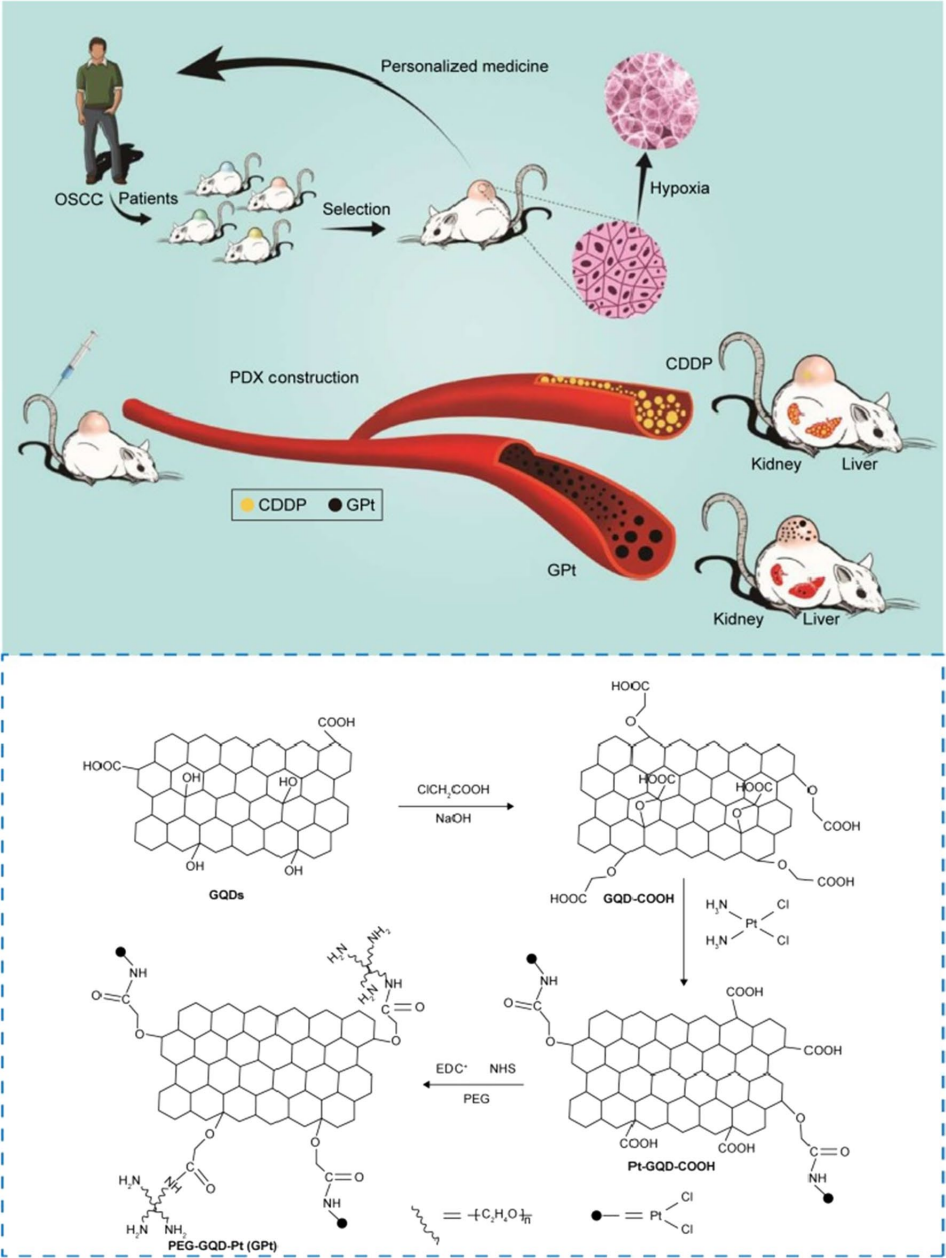
Schematic illustration of a multifunctional platform for anticancer therapy with high efficacy against hypoxia-induced chemoresistance of OSCC (reprinted/reproduced with the permission of Ref. [189], (reprinted/reproduced with the permission of Ref. [189], copyright 2018, Int J Nanomedicine)

To overcome the hypoxia-induced resistance to chemotherapy in the tumor microenvironment, Wei et al. [189] established a DDRS based on Pt and polyethylene glycol-GQDs. First, GQDs were synthesized by chemical oxidation of dried CX-7 carbon black with HNO_3_. Subsequently, ClCH_2_COH hydroxylated GQDs-COOH supported cisplatin via a covalent bond. Further, Pt-GQDs-COOH was obtained. Finally, polyethylene glycol (PEG) was connected to Pt-GQDs-COOH by stirring to synthesize a polyethylene glycol-graphene quantum dot-Pt (GPt) with a diameter of about 5 nm (Fig. 13). Oral squamous cell carcinoma (OSCC) and BALB/cJNJu-Foxn1^nu^/Nju (4 weeks old) xenograft tumor male mice were used to detect GPt in vitro and in vivo, respectively. It was found that GPt has a good therapeutic effect on OSCC under both normoxic and hypoxic conditions. Compared with free cisplatin, after GPt enters mice through the tail vein, GPt has a stronger inhibitory effect on tumor growth, and systemic drug toxicity is not obvious. This is mainly because GPt is easier to enter tumor tissues through the EPR effect, and releases Pt for anti-tumor in an acidic environment. Potential new strategies for the preparation of GPt for therapy targeting the tumor microenvironment.

**Fig. 14:**
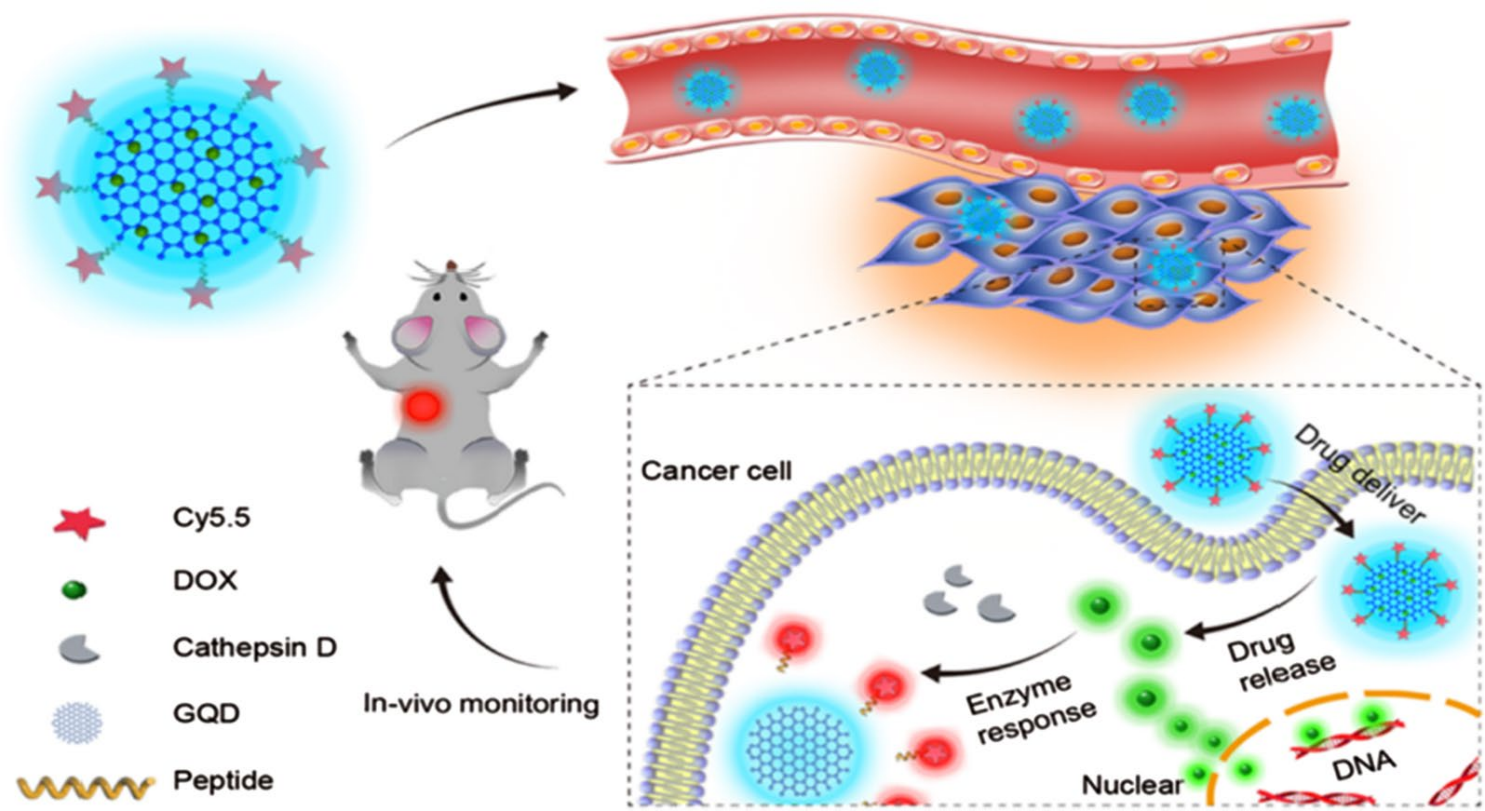
Strategy of GQDs-based theranostic agent for programmatically monitoring anticancer drug delivery, release, and response (reprinted/reproduced with the permission of Ref. [41], copyright 2017, ACS Applied Materials & Interfaces)

In general, when the function of DDRS is usually relatively simple, in this EPR-pH delivery-release mode, the drug may be released early during delivery, and the efficacy will be reduced due to the lower penetration performance. To improve tissue permeability and cell uptake, Ding et al. [41] developed a new type of anticancer drug with excellent therapeutic properties. Firstly, GQDs were prepared from polyacrylonitrile carbon fibers by simple chemical oxidation and exfoliation. DOX is then loaded on the surface of GQDs via π–π interaction to obtain DOX@GQDs. GQDs were coupled with Cy5.5 (Cy) dye, a NIR fluorescent molecule, via a cathepsin D-responsive (P) peptide, and finally DOX@GQDs-P-Cy was synthesized. DOX@GQDs-P-Cy was evaluated in vitro and in vivo by 4T1 breast cancer cells, 3D multicellular tumor spheroid (MCTS) model, and tumor-bearing mice, respectively (Fig. 14). It was found that GQDs-P-Cy has good biocompatibility. After loading DOX, it is significantly more cytotoxic than free DOX, and DOX@GQDs-P-Cy has stronger tumor penetration ability. DOX@GQDs-P-Cy is injected intravenously into tumor-bearing mice. Compared with free DOX, tumor uptake of DDRS is more favorable for tumors. The tumors shrink faster, and tumor-bearing mice survive longer. In addition, the DOX@GQDs-P-Cy can also be used as a probe to trace the delivery process and release site of anticancer drugs. The negative correlation between the fluorescence intensity of the NIR fluorescence signal of Cy triggered by cathepsin D and the relative speed of tumor growth can also be used to accurately assess the apoptosis in chemotherapy in real time. This versatile DDRS that monitors drug delivery, release, and treatment will help establish personalized anti-cancer therapies.

### Ligand-pH delivery-release mode

Generally, to achieve accurate anti-tumor treatment, first, the antitumor drugs are loaded on DDRS through π–π interactions. Then, tumor is targeted by the DDRS-loaded drugs through ligand-receptor interactions. Finally, antitumor drugs are released from DDRS in the low pH environment of tumor cells for effective tumor ablation [190].

**Fig. 15:**
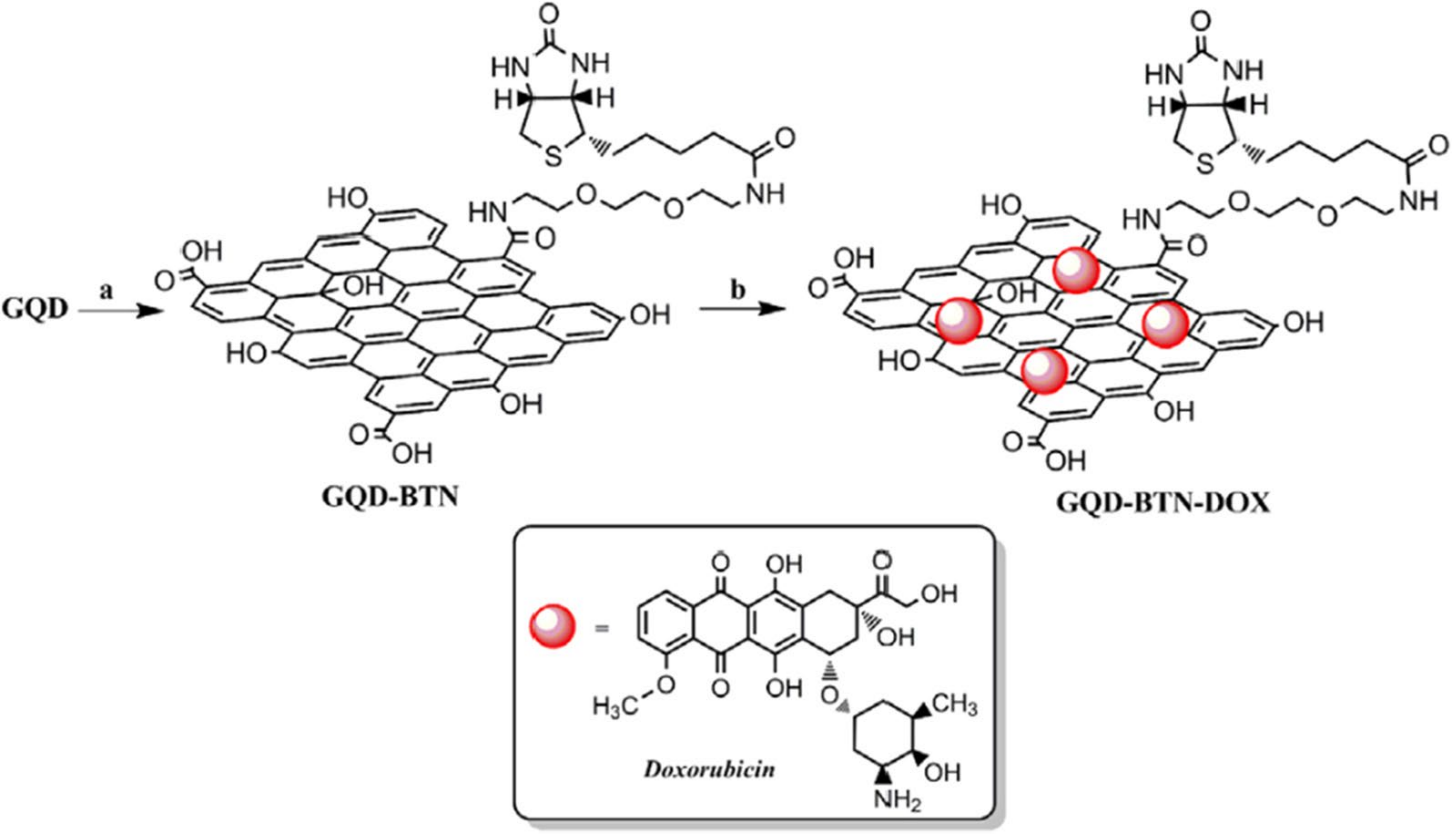
Synthesis of GQDs-BTN-DOX. Reagents and conditions: **a** BTN, EDC·HCl, HOBt, DMAP, CH_2_Cl_2_, 4d, r.t.; **b** DOX, buffer solution pH 7.4, 24 h, r.t (reprinted/reproduced with the permission of Ref. [191], copyright 2017, Int J Pharm)

Iannazzo et al. [191] established a good biocompatible and cell-traceable drug delivery system based on GQDs for inserting the doxorubicin (DOX) drug into DNA for delivery to cancer cells (Fig. 15). They first synthesized highly dispersed water-soluble GQDs by chemical oxidation using MWCNTs as raw materials. In order to be able to effectively recognize biotin receptors that are overexpressed in cancer cells, they are covalently linked to the tumor targeting module biotin (BTN) and loaded with DOX through π–π interactions to obtain GQDs-BTN-DOX delivery-release system. In vitro cytotoxicity tests using A549 cells showed that the synthesized GQDs and GQDs-BTN had no significant toxicity. In A549 cells treated with GQDs-BTN-DOX, cytotoxicity is closely related to cellular uptake. After treatment with GQDs-BTN-DOX, cell uptake was larger and delayed compared to cell uptake observed with GQDs-DOX or free DOX. The drug delayed nuclear internalization because the acidic environment of cancer cells caused the drug to detach from the system. In addition, intrinsic fluorescence enables tracking of drug release.

**Fig. 16:**
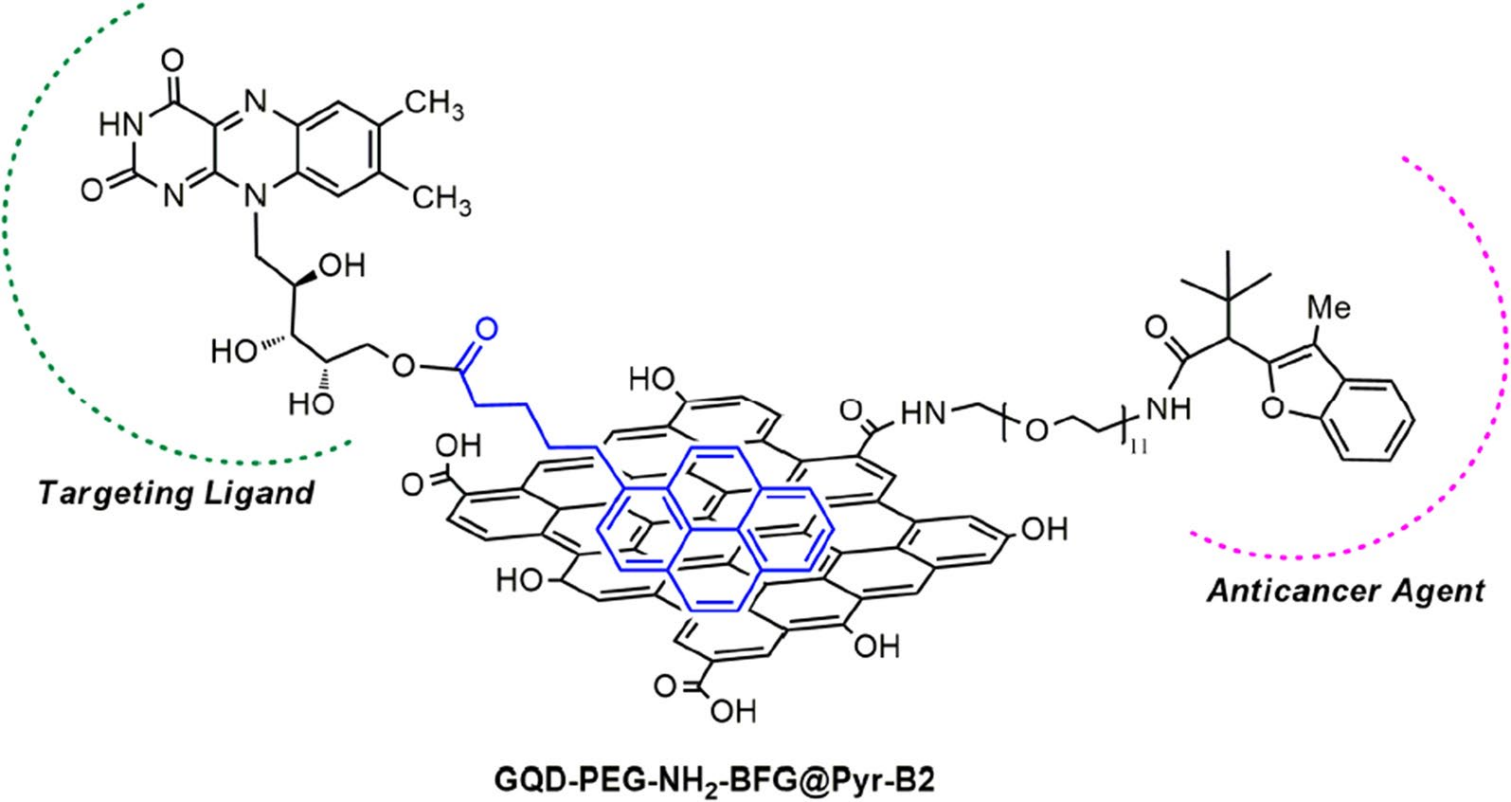
Cancer targeted DDRS based on GQDs (reprinted/reproduced with the permission of Ref. [192], copyright 2019, Nanomaterials)

Based on the previous experience, they [192] have prepared similar intelligent DDRS, which also has drug loading and targeting functions. First, GQDs and PEG are assembled to form GQDs-PEG-BFG, which has the function of supporting anticancer drugs with a benzofuran structure (BFG). Then, using pyrene as a linker, it was linked to the targeting ligand riboflavin (RF, vitamin B2) through a π–π interaction. The GQDs-PEG-BFG@Pyr-RF (Fig. 16) was formed to target cancer cells. Functions. Biological tests were performed on synthetic DDRS using three cancer cell lines, laryngeal cancer cell line (HEp-2), human lung epithelial cancer cell line (A549), and human colorectal adenocarcinoma cell line (HT-29). GQDs-PEG-BFG@Pyr-RF, GQD-PEG-BFG, and GQDs@Pyr-RF were found to be low cytotoxic, but toxic to cancer cells, compared to free BFG. This new DDRS provides new ideas for the application of anticancer drugs with poor water solubility, low cell uptake, systemic toxicity and adverse side effects. It also shows that GQDs, a new generation member of the graphene family, have shown broad application prospects in anticancer treatment.

**Fig. 17:**
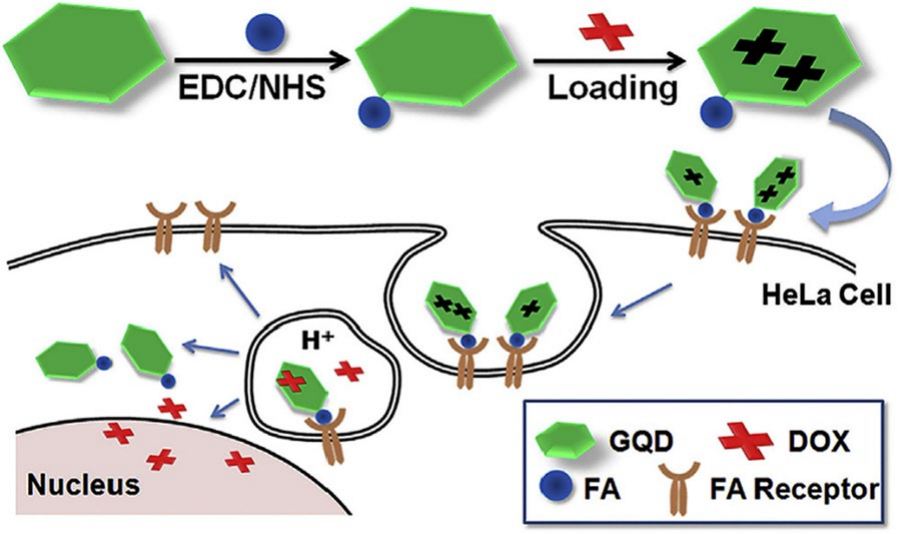
Schematic of the fabricated DOX-GQDs-FA nanoassembly for DOX deliveryinto target cells (reprinted/reproduced with the permission of Ref. [194], copyright 2014, Colloids and Surfaces B: Biointerfaces)

Qin et al. [193] developed a new nano-scale DDRS based on GQDs, only 9–12 nm, for the treatment of ovarian cancer. First, GQDs were synthesized by hydrothermal method using graphite as a raw material. Subsequently, the targeting ligand folic acid (FA) was coupled to GQDs, giving GQDs the ability to target cancer cells. Next, the chemotherapeutic DOX is loaded on it via π–π stacking interaction to form the final DDRS, GQDs-FA-DOX, which was somewhat similar to the design by Wang et al. [194] (Fig. 17). The performance of GQDs-FA-DOX was tested using the normal ovarian epithelial cell line T80 and the ovarian carcinoma cell line OVCAR3. It was found that the synthesized DDRS had no toxic effect on normal ovarian cells and had ideal therapeutic effects on ovarian cancer cells. GQDs-FA-DOX provides a novel and effective strategy for targeted therapy of ovarian cancer.

**Fig. 18:**
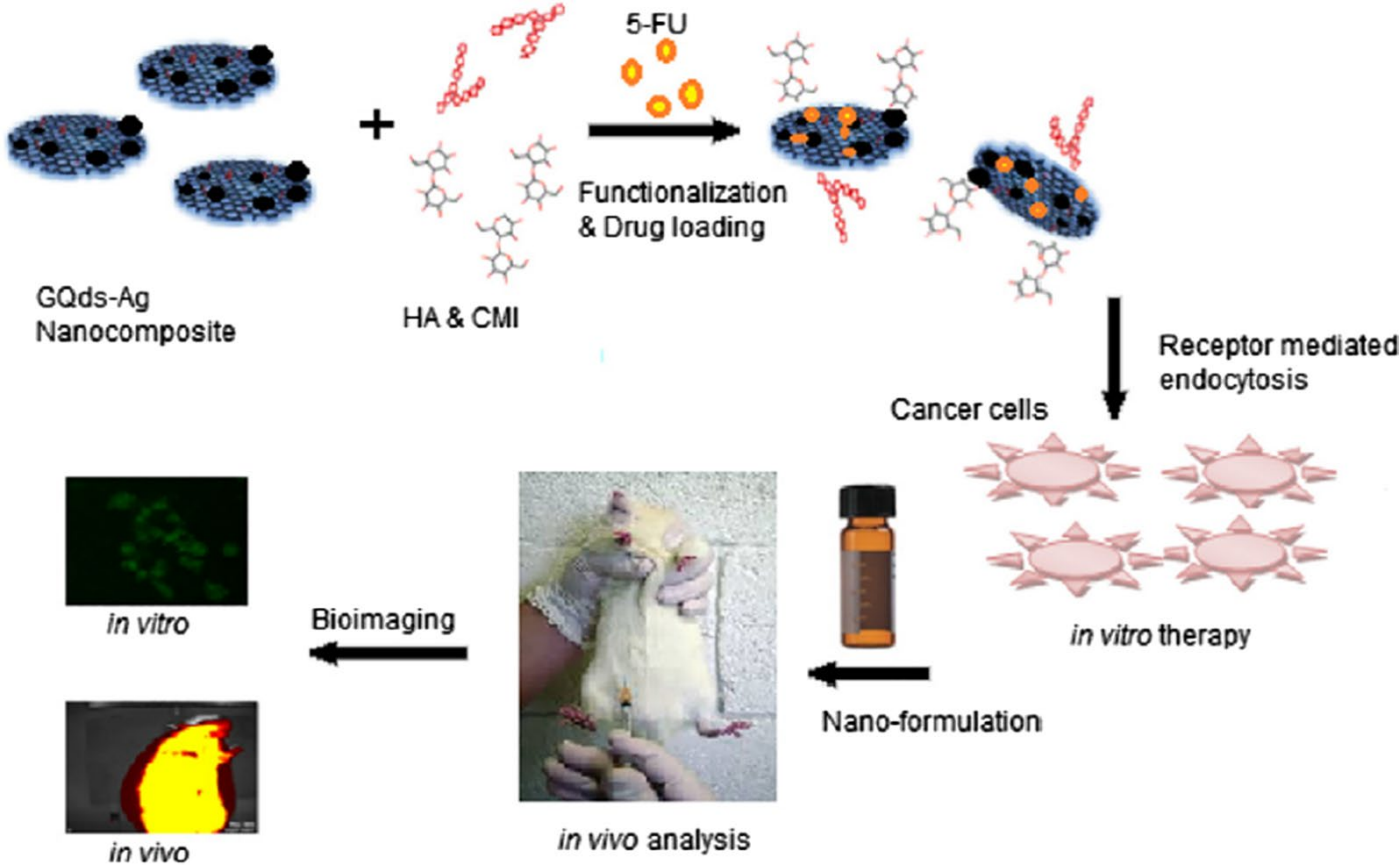
Schematic diagram of nanoformulation preparation and in vitro and in vivo theranostic applications (reprinted/reproduced with the permission of Ref. [195], copyright 2017, Materials Science and Engineering: C)

In addition, in order to deliver pancreatic cancer-specific drugs in rats, Joshi et al. [195] synthesized a novel DDRS (Fig. 18) based on silver-graphene quantum dot (Ag-GQDs) nanocomposites. First, glutamine was used as the raw material, and pure GQDs were obtained after high-temperature cracking at 190–200 °C and further high-speed centrifugation to remove impurities. Subsequently, GQDs and a certain amount of AgNO_3_ solution were mixed, and Ag^+^ was reduced in situ using tri sodium citrate as a reducing agent to obtain an Ag-GQDs nanocomposite. In order to reduce the toxicity of silver nanoparticles and improve the biocompatibility of nanoparticles, carboxymethyl inulin (CMI), a modification of natural polysaccharide inulin, is coupled with carbodiimide and nanocomposites to obtain Ag-GQDs-CMI. Hyaluronic acid (HA), as a target component of CD-44 (cancer stem cell marker), is connected to Ag-GQDs through an EDC-NHS coupling reaction, so that the synthesized HA-Ag-GQDs-CMI can reach cancer cells accurately. ability. 5-Fluorouracil (5-FU), as a model drug, is loaded on it by adsorption and released in the acidic environment of cancer cells. After in vitro biological characterization of three cell lines—HeLa (Cervical cancer), HepG2 (Liver cancer), and Panc-1 (Pancreatic cancer)—in vitro and in Wistar rats, a synthetic 5-FU-containing DDRS was found (72–78% inhibition) inhibited cancer cells more effectively than Ag-GQDs-CMI (~ 56%) with 5-FU and had stronger anti-cancer effect. This shows the potential promise of Ag-GQDs-CMI in targeted delivery-release drugs.

The most commonly used delivery-release mode is the ligand-pH delivery-release mode. Synthetic DDRS is typically nano-sized and reaches the tumor site through blood flow. Due to the unique ligand-receptor binding, drug delivery is accurate. However, because the release is pH-mediated, the drug is sometimes lost during the delivery, which leads to a decrease in the treatment efficiency.

### EPR-photothermal delivery-release mode

In the EPR-photothermal delivery-release mode, DDRS can be two-dimensional (2D) or three-dimensional (3D), but because it lacks a ligand and does not have a targeting function, it has no magnetic iron oxide and is not controlled by a magnetic field. Generally, it can only be accumulated at the tumor site through the EPR effect, but the release of DDRS can be controlled by NIR radiation without relying on the tumor’s slightly acidic environment.

**Fig. 19:**
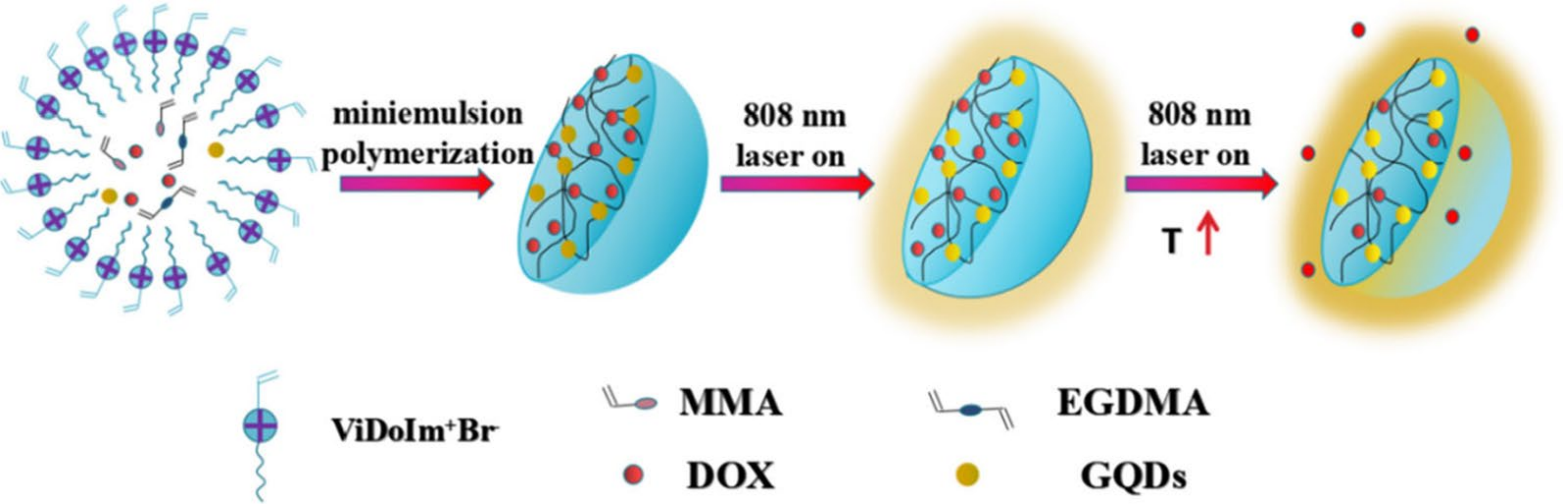
Schematic diagram of GMIPs synthesis and DOX release by an ex vivo (reprinted/reproduced with the permission of Ref. [123], copyright 2019, Journal of Materials Science)

Xu et al. [123] reports a DDRS that can reduce the drug leakage and improve drug release efficiency in tumor lesions (Fig. 19). First, polymerizable ionic liquid ViDoIm^+^Br^−^ as an emulsifier successfully prepared polymer microsphere including GQDs, DOX, MMA, and EGDMA via miniemulsion polymerization. The study found that compared with non-imprinted polymers (GNIPs), molecularly imprinted polymers (GMIPs) greatly improved the loading efficiency of DOX and reduced drug leakage. DDRS can accumulate in tumor areas through the EPR effect. In addition, because GQDs have a light-to-heat conversion effect, DOX that releases the load from GMIPs can be controlled by NIR radiation. The combination of molecular imprinting technology and photothermal controllable drug delivery system provides an idea for designing novel DDRS that can reduce drug loss and improve drug release efficiency.

**Fig. 20:**
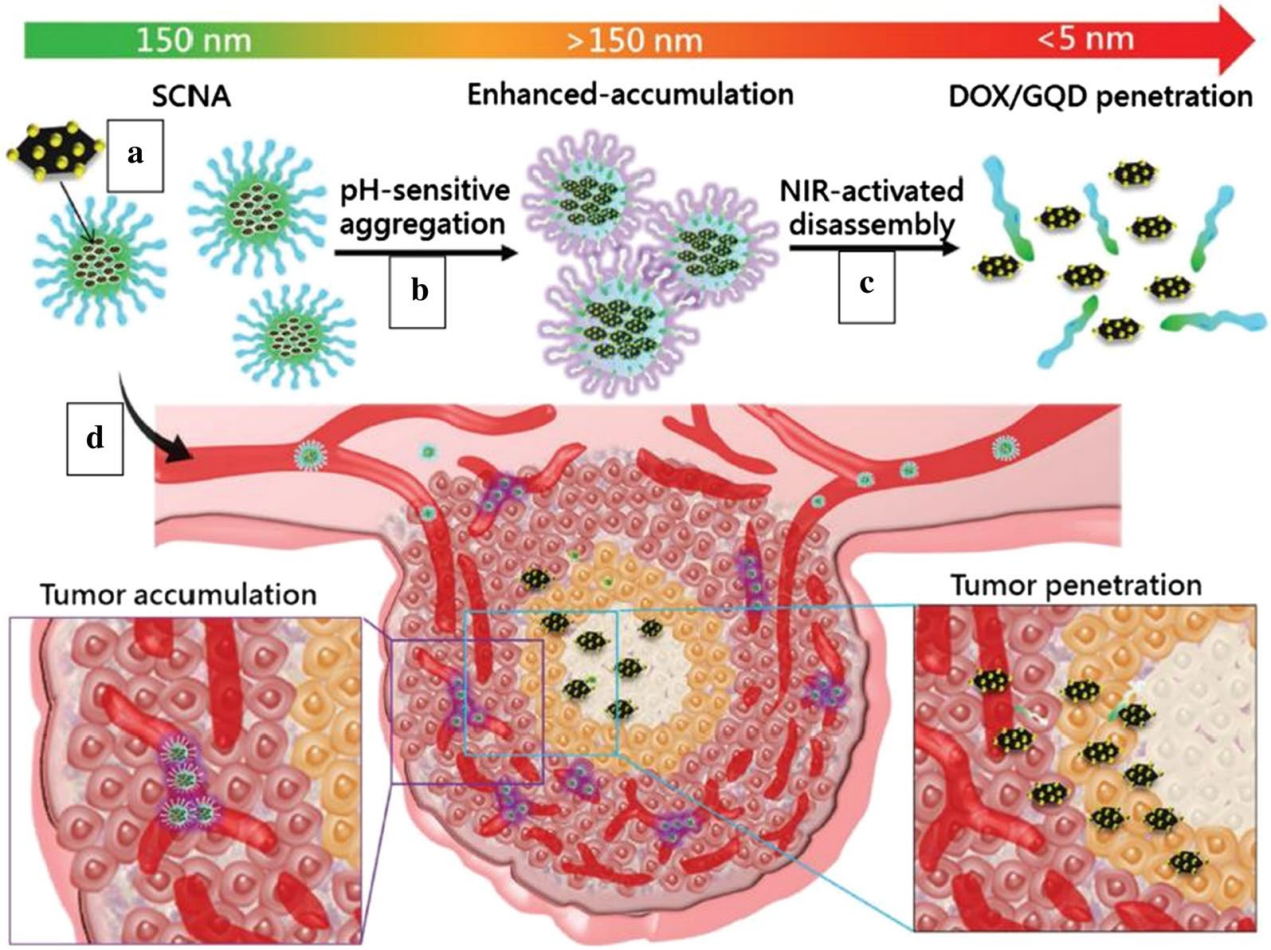
Size-changeable nanoaircrafts (SCNAs) for hierarchical tumor targeting through an aggregation transition in the weak acidity of the tumor environment and photopenetrating drug/GQDs delivery. **a** The SCNAs delivered DOX/GQDs to the tumor through intravenous injection. **b** The aggregation transition of the SCNAs in the weak acidity of the tumor environment enhanced tumor accumulation. **c** NIR-activated disassembly of SCNAs into DOX/GQDs facilitated penetration deep into tumors. **d** Schematic illustration of the enhanced tumor accumulation and penetration by SCNAs (reprinted/reproduced with the permission of Ref. [196], (reprinted/reproduced with the permission of Ref. [196], copyright 2017, Advanced Functional Materials)

Aiming at the targeting and penetration of deep tumors, Su et al. [196] has synthesized a pH-sensitive GQDs nanoaircraft (SCNA) with variable size and NIR-mediated drug release, which can deliver and penetrate antitumor drugs into the deep Tumor (Fig. 20). Firstly, using hexaphenylbenzene as a starting material, artificial graphite (AG) was synthesized by a bottom-up method. Subsequently, Hummers method was used to prepare artificial graphene oxide (AGO) from AG, followed by ultrasonic dispersion of the AGO solution containing hydrazine to obtain GQDs. Finally, GQDs loaded with DOX through π–π stacking interactions self-assembled into SCNA with HTPGS containing a hydrophilic PEG strain and a hydrophobic vitamin E via hydrophobic interaction. The SCNA is injected into RG2 tumor-bearing BALB/c nude mice via intravenous injection and has a stealth function due to stability at physiological pH. When the SCNA entered the weak acidity of tumor environment through the enhanced permeability and retention (EPR) effect, it exhibited an aggregation transition, which expanded from 150 to 450 nm (in vitro pH 6.6). When further driven by NIR, SCNA breaks down into DOX/GQDs (5 nm) and penetrates deep tumor tissues like a bomb-loaded jet. It is exciting that DOX/GQDs that penetrate tumor tissue can infect and repeatedly kill adjacent tumor cells without causing damage to the distal end. This nanocomposite with enhanced tumor permeability combined with photothermal chemotherapy is expected to open a new way to overcome the limitations of nanoparticles in tumor treatment.

Compared with the EPR-pH delivery-release mode, the EPR-photothermal delivery-release mode goes one step further. Instead of relying solely on the tumor’s micro-acid environment, it has a light-to-heat conversion capability and can control the release of DDRS through NIR radiation. This approach reduces the early release of drugs and improves the efficiency of drug treatment.

### Core/Shell-photothermal/magnetic thermal delivery-release mode

Some researchers [187, 188] have developed core–shell materials, which are generally combined with photodynamic therapy and thermodynamic therapy, providing new ideas for drug delivery and release.

**Fig. 21:**
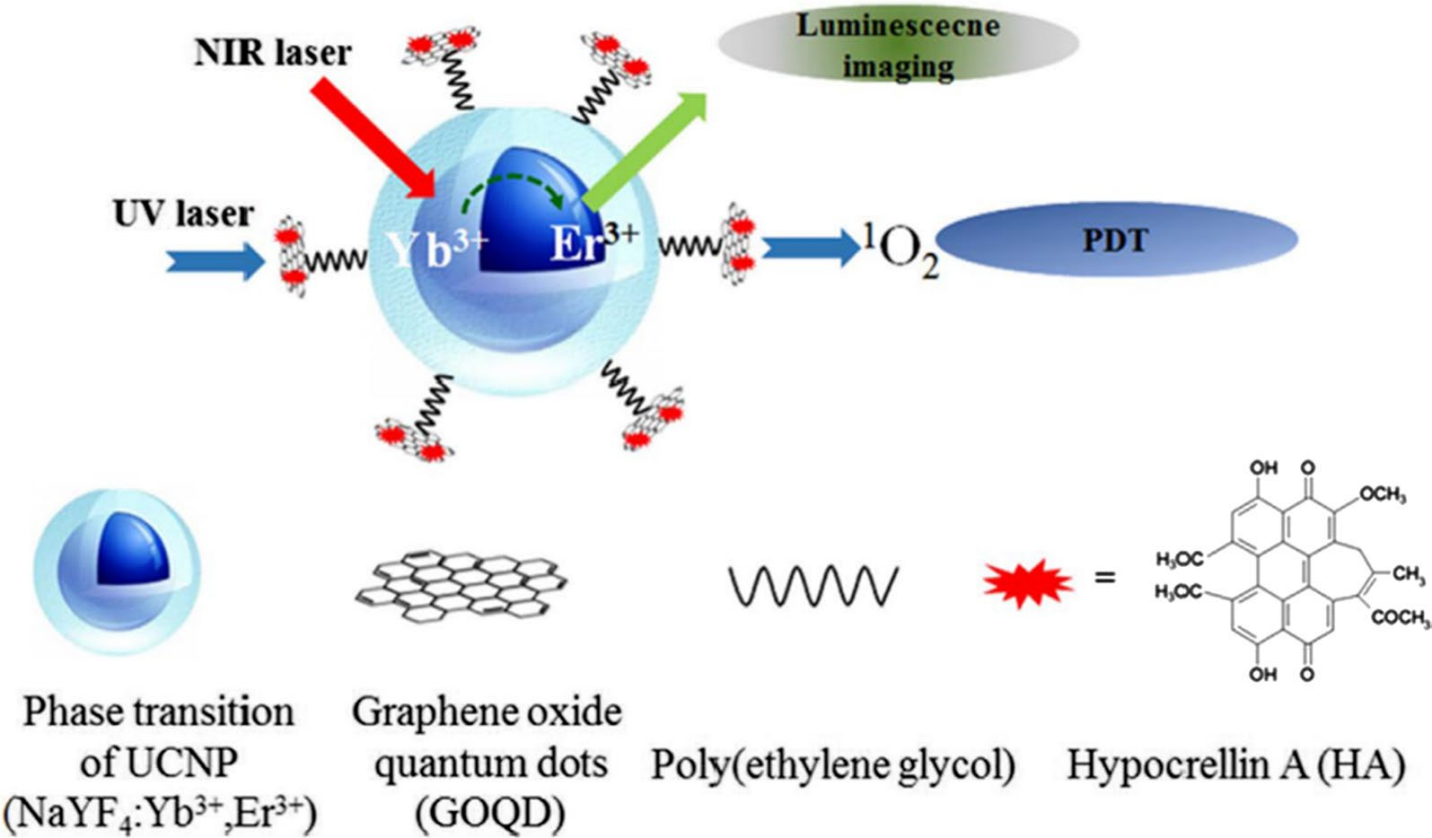
The action procedure of HA/GOQD/UCNP nanoparticle (reprinted/reproduced with the permission of Ref. [197], copyright 2017, Biosensors and Bioelectronics)

With upconversion nanoparticle (UCNP) as the core and GOQDs as the shell, Choi et al. [197] also synthesized a core–shell nanoparticle for cell imaging and drug delivery. Firstly, UCNP (NaYF_4_:Yb3^+^, Er3^+^) was synthesized by hydrothermal method, and the surface of UCNP was further modified by polyethylene glycol 2-aminoethyl ether acetic acid (amine-PEG). GOQDs were coupled to Hypocrellin A (HA), a commonly used chemotherapeutic drug and photosensitizer, through π–π interaction and loaded onto the surface of UCNP via PEG to synthesize HA/GOQDs/UCNP core–shell structure (Fig. 21). HeLa cells were used to study HA/GOQDs/UCNP in vitro. The study found that GOQDs/UCNP and HA/GOQDs had no significant cytotoxicity regardless of whether they were irradiated (460 nm LED). Compared with the non-irradiated group, the cell activity of the HA/GOQDs/UCNP irradiated group was significantly reduced (52%), indicating that HA/GOQDs/UCNP irradiation at 460 nm improved the anti-tumor treatment effect. Upconversion luminescence (UCL) imaging shows that HA/GOQDs/UCNP is an effective biological probe that can be used for cell imaging. Synthetic core–shell structured nanoparticles are potential candidates for multifunctional agents for cell imaging, drug delivery, and cell therapy.

**Fig. 22:**
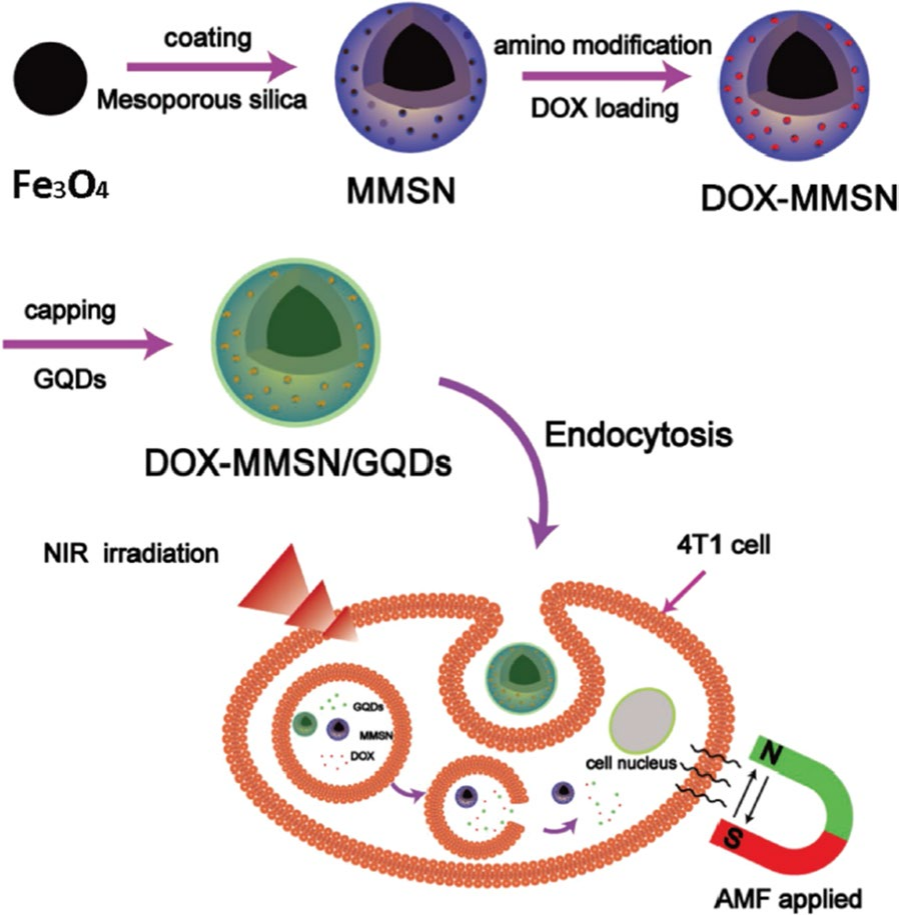
Schematic illustration of the preparation process of the DOX-MMSN/GQDs nanoparticles and synergistic therapy combined with controlled drug release, magnetic hyperthermia, and photothermal therapy (reprinted/reproduced with the permission of Ref. [141], copyright 2016, Small)

Yao et al. [141] established a multifunctional platform for drug release, magnetic thermotherapy and photothermotherapy, consisting of caps and local photothermal generators, drug carriers and magnetic thermoseeds. Firstly, magnetic mesoporous silica nanoparticles (MMSN), drug carriers and magnetic thermoseeds were synthesized by sol–gel process with magnetic Fe_3_O_4_ nanoparticles prepared by solvothermal method as the core. Next, APTES-functionalized amine-modified MMSN absorbed DOX by stirring in the dark for 24 h. Then, DOX-MMSN and the activated GQDs were mixed at 4 °C in the dark for DOX encapsulation. *N*-Hydroxysuccinimide (NHS) and 1-ethyl-3-(3-dimethylaminopropyl) carbodiimide hydrochloride (EDC) activate GQDs as caps and local photothermal generators. Finally, a core–shell structure of MMSN/GQDs DDRS with a diameter of 100 nm was obtained (Fig. 22). In vitro biological detection of MMSN/GQDs DDRS was carried out using breast cancer line 4T1 cells as model cell system. It was found that the release of DOX in DOX-MMSN/GQDs could be induced in a low pH environment to perform chemotherapy of tumor cells. In addition, this DDRS can effectively heat cells to high temperature under alternating magnetic fields or NIR radiation for magnetic or photothermal treatment of tumor cells. Compared with incomplete DDRS treatment, the combined use of chemotherapy, magnetocaloric therapy, or photothermal therapy has a significant synergistic effect greatly improving the anti-tumor efficiency. Therefore, this multi-functional platform (MMSN/GQDs DDRS) that enhances anti-tumor efficiency has a great potential application in anti-tumor treatment.

**Fig. 23:**
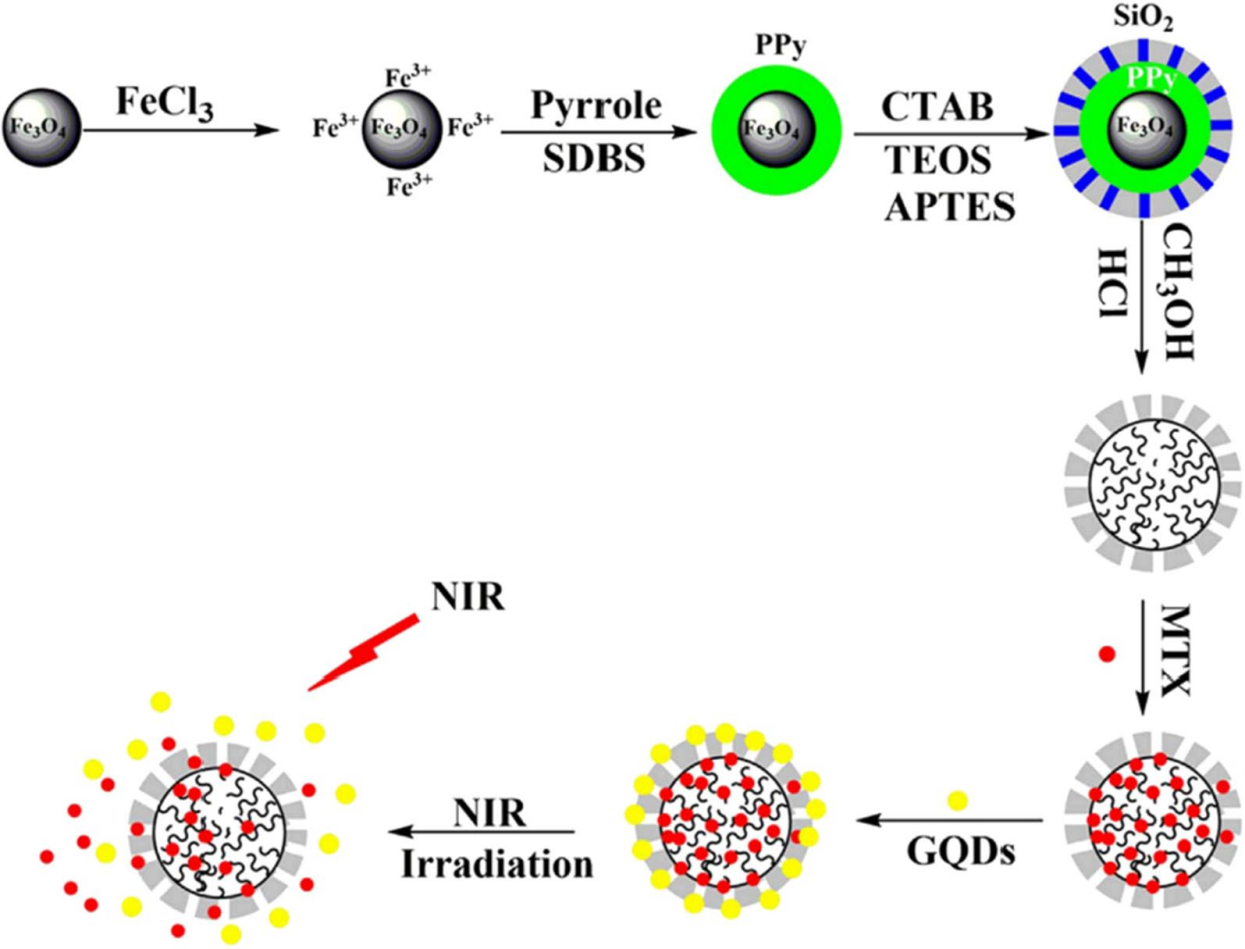
Schematic illustration showing the synthesis of PPy/mSiO_2_, the encapsulation of MTX with the aid of GQDs cap and the NIR light-triggered MTX delivery (reprinted/reproduced with the permission of Ref. [198], copyright 2017, Materials Science and Engineering: C)

In order to reduce drug loss during transportation, Li et al. [198] synthesized polypyrrole/mesoporous SiO_2_/GQDs (PPy/mSiO_2_/GQDs) core–shell nanocomposites (Fig. 23) for drug delivery. Firstly, using Fe_3_O_4_ as the core, Ppy-Fe_3_O_4_ was prepared. Polypyrrole (PPy), has a very high light-to-heat conversion efficiency at 808 nm and shows strong absorption in the NIR region. Subsequently, a mesoporous SiO_2_ layer was formed on the surface of PPy–Fe_3_O_4_, and Fe_3_O_4_ was removed to form a PPy/mSiO_2_ core–shell structure. After stirring the MTX load, GQDs were introduced into the outer surface of PPy/mSiO_2_, forming hydrogen bonds with the outer surface of mSiO_2_, thereby packaging the drug to prevent the drug from being lost during transportation. Under NIR radiation from the tumor site, PPy converts light into heat. As the temperature of PPy/mSiO_2_/GQDs increased, the hydrogen bonds between GQDs and mSiO_2_ were broken and fell off. MTX was released from PPy/mSiO_2_ for antitumor treatment. The intelligent drug delivery-release of PPy/mSiO_2_/GQDs could provide a new mechanism for medical diagnosis and treatment.

Loading the antitumor drug into the core–shell structure can effectively prevent the early release of drug during the delivery. Under the irradiation of light or applying magnetic field, the cell penetration efficiency and cell absorption capacity of nano-sized core–shell structure could be significantly improved, thereby improving the tumor treatment efficacy. In addition to chemotherapy, photothermal therapy and magnetocaloric therapy have also been developed, and with extensive studies by researchers, multiple therapeutic approaches in conjunction with antitumor have gradually become a trend for tumor therapy [144].

### Other delivery-release modes

Some researchers have also developed other delivery-release modes using GQDs. In addition to synthesizing nanoscale DDRS for intravenous administration, GQDs can also be used for micro and millimeter DDRS for oral administration. In order to avoid the trauma and discomfort caused by intravenous or other methods of administration, for some diseases, oral delivery systems have also attracted much attention.

**Fig. 24:**
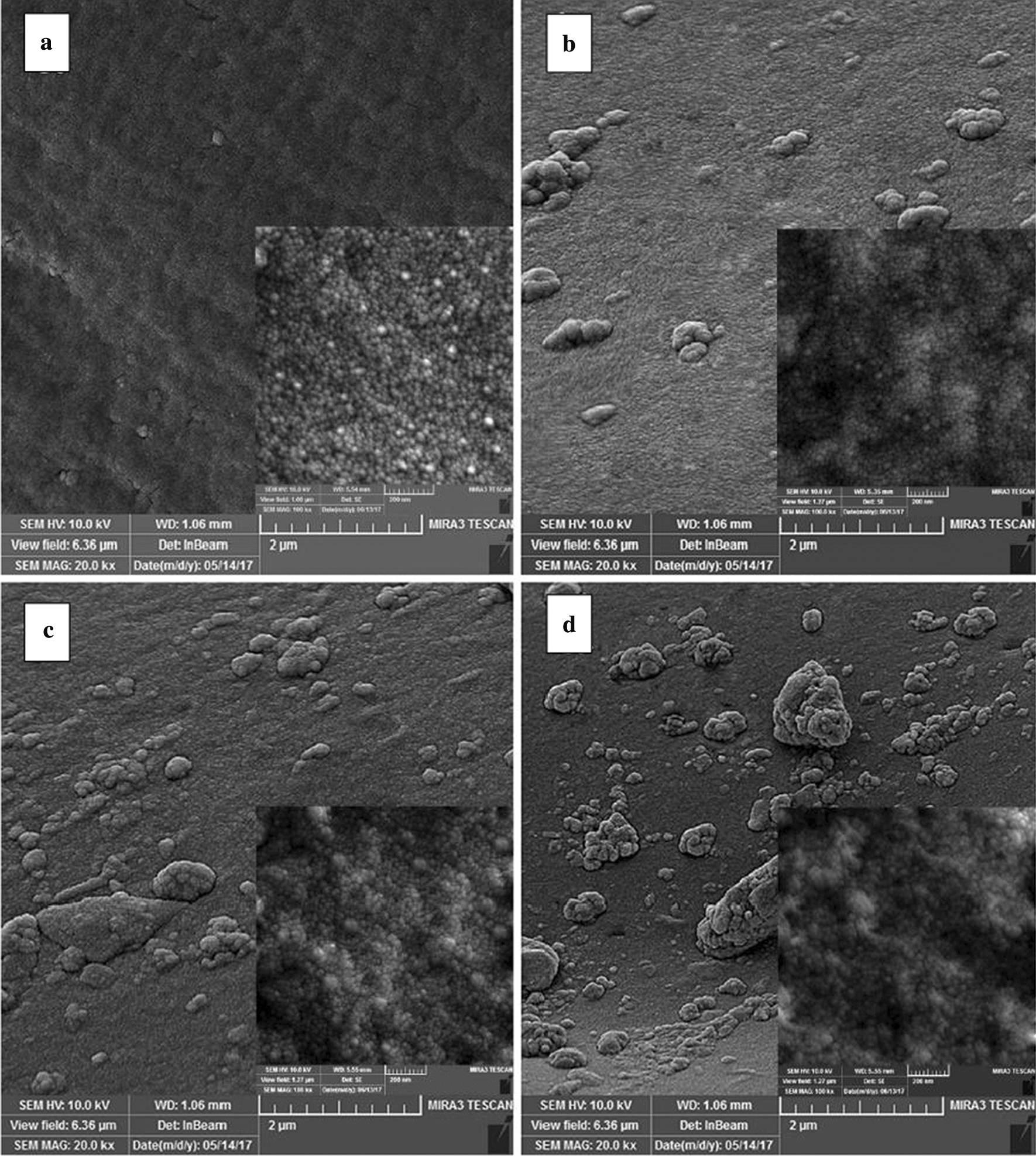
SEM images of the **a** Cu-CMC/NPX and Cu-CMC/NPX/GQDs, **b** 10%, **c** 20%, **d** 30% GQDs content (insets are higher magnifications) (reprinted/reproduced with the permission of Ref. [199], copyright 2018, Carbohydrate polymers)

**Fig. 25:**
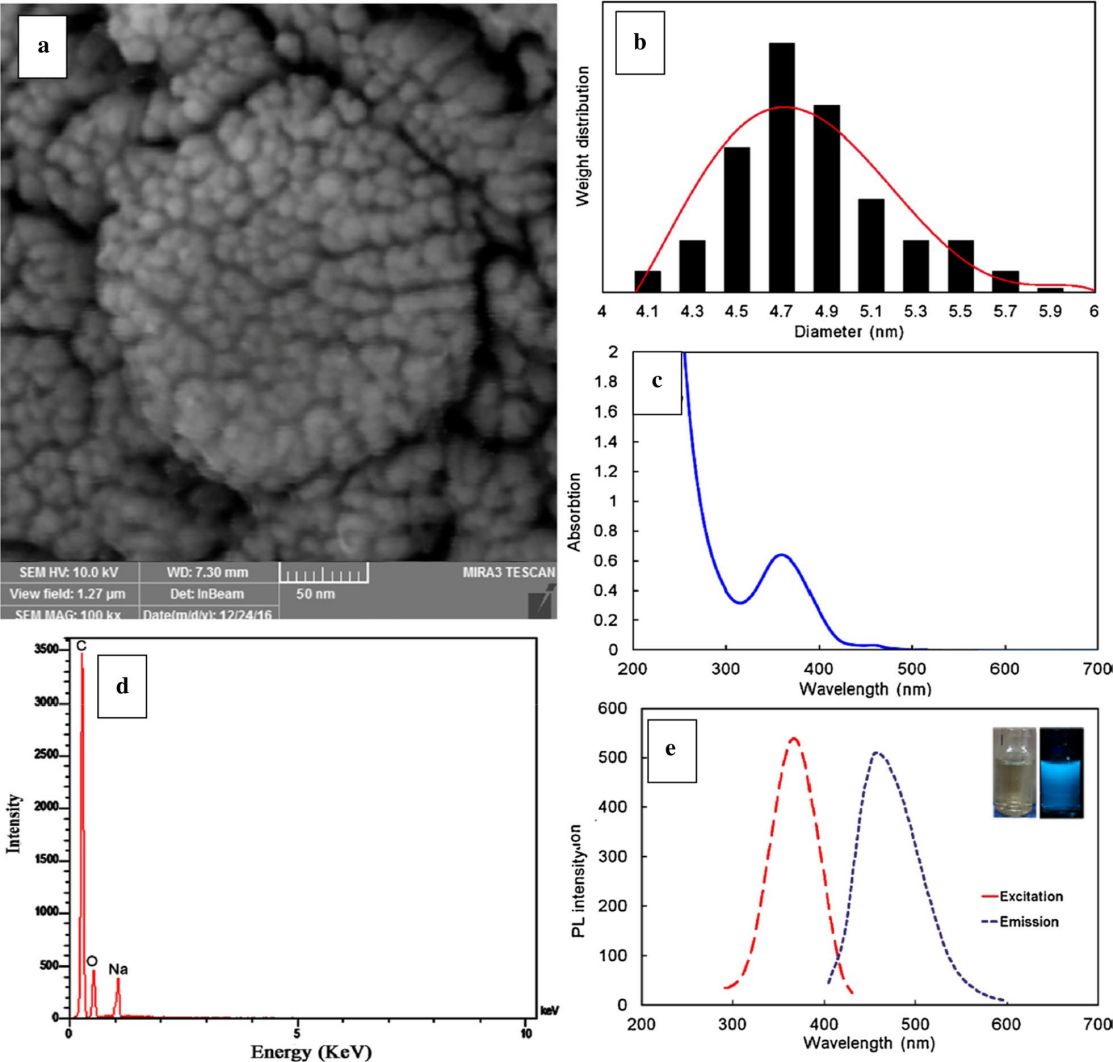
Characterization of GQDs: **a** SEM, **b** DLS of GQDs, **c** UV–Vis absorption spectra of GQDs in water, **d** EDX analysis for the prepared GQDs, **e** PL spectrum of the dilute aqueous solution of GQDs and the photograph demonstrates the fluorescence of a dilute aqueous solution of GQDs (reprinted/reproduced with the permission of Ref. [199], copyright 2018, Carbohydrate polymers)

Hydrogel nanocomposite microspheres prepared based on carboxymethylcellulose (CMC) have received widespread attentions due to their many advantages, such as mild, simple, compact, and uniform shape to be used for drug delivery and release. Therefore, Javanbakht et al. [199] synthesized hydrogel nanocomposite microspheres based on CMC and GQDs for oral administration (Fig. 24). Firstly, CA prepared GQDs by pyrolysis, and then pre-loaded the model drug naproxen (NPX) on the surface of GQDs by stirring to form GQDs-NPX (Fig. 25) [113]. CMC was further added, and copper acetate was added as a physical cross-linking agent under continued stirring. Finally, Cu-crosslinked carboxymethylcellulose/naproxen/graphene quantum dots nanocomposite hydrogel beads (Cu-CMC/NPX/GQDs) were obtained after drying at room temperature. After simulating oral administration, the release of the drug in the gastrointestinal tract found that CMC can effectively protect the loaded drug from the effects of a low pH environment. By controlling the release of different pH environments, the long-term effects of the drug can be extended. Caco-2 cells was used to detect the cytotoxicity of Cu-CMC/NPX/GQDs. It was found that the prepared hydrogel nanocomposite beads had no obvious toxicity and could be used as drug capsules to form an oral drug delivery-release system in the gastrointestinal environment.

**Fig. 26:**
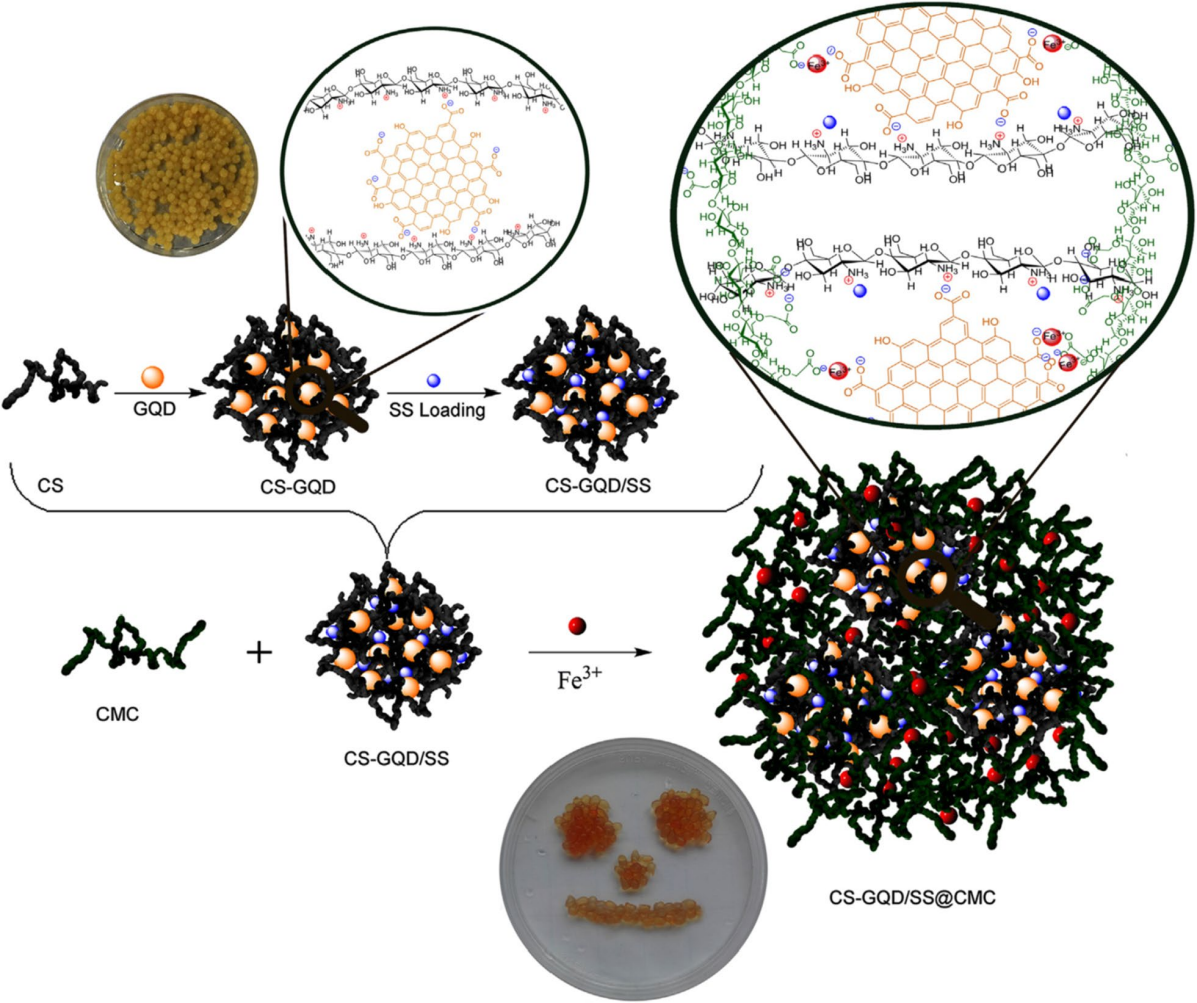
The schematic representation of coating of CS-GQDs/SS with CMC and preparation of CS-GQDs/SS@CMC bio-nanocomposite hydrogel beads (reprinted/reproduced with the permission of Ref. [200], (reprinted/reproduced with the permission of Ref. [200], copyright 2019, International Journal of Biological Macromolecules)

**Fig. 27:**
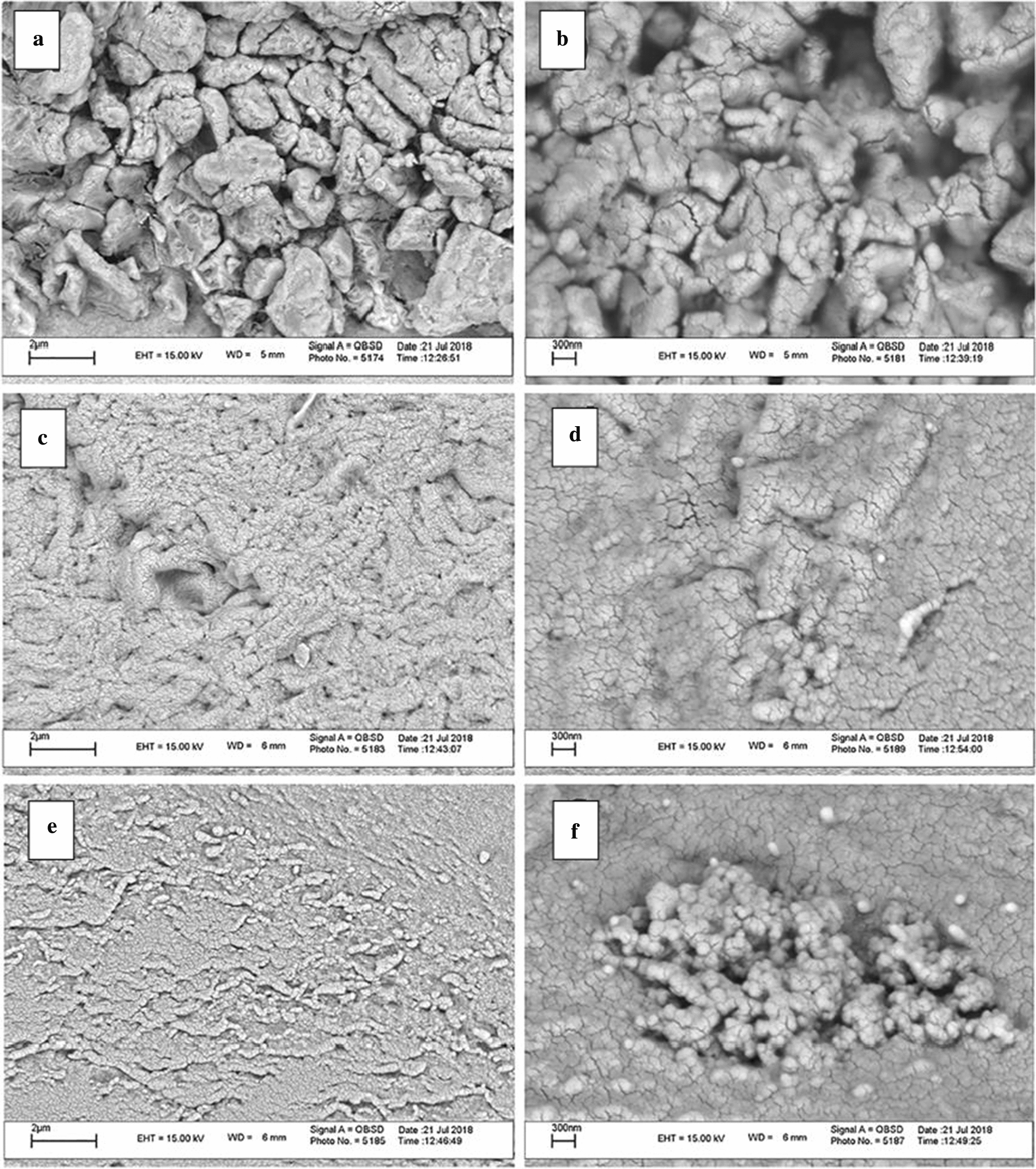
SEM images of the **a** CS-GQDs, **c** CMC@SS, **e** CS-GQDs/SS@CMC at low magnification and SEM images of the **b** CS-GQDs, **d** CMC@SS, **f** CS-GQDs/SS@CMC at high magnifications (reprinted/reproduced with the permission of Ref. [200], copyright 2019, International Journal of Biological Macromolecules)

Subsequently, they [200] developed new bio-nanocomposite beads for oral administration. First, using the previously prepared GQDs [113, 199] as a cross-linking agent, the beads of chitosan (CS) acetate solution prepared by a syringe (2 mm diameter) were cross-linked. CS-GQDs bio-nanocomposite hydrogel beads were obtained after vacuum drying. Afterward, CS-GQDs were loaded with SS by immersing in the model drug sodium salicylate (SS) solution to form CS-GQDs/SS. Next, CS-GQDs/SS was encapsulated and protected by pH-sensitive CMC by means of stirring and syringe extrusion, and finally Fe^3+^ cross-linking was carried out with ferric chloride hexahydrate solution. After drying, the CMC encapsulated CS-GQDs/SS bio-nanocomposite hydrogel beads (CS-GQDs/SS@CMC) were synthesized (Fig. 26). After simulated oral administration in vitro, it was found that the prepared beads were stable under the conditions of the gastrointestinal tract and could prolong the stability of administration for a long time (Fig. 27). MTT test of human colon adenocarcinoma HT29 cells showed that the bio-nanocomposite beads had low cytotoxicity. In conclusion, the new CS-GQD/SS@CMC is expected to potentially serve as a safe carrier for oral administration.

**Fig. 28:**
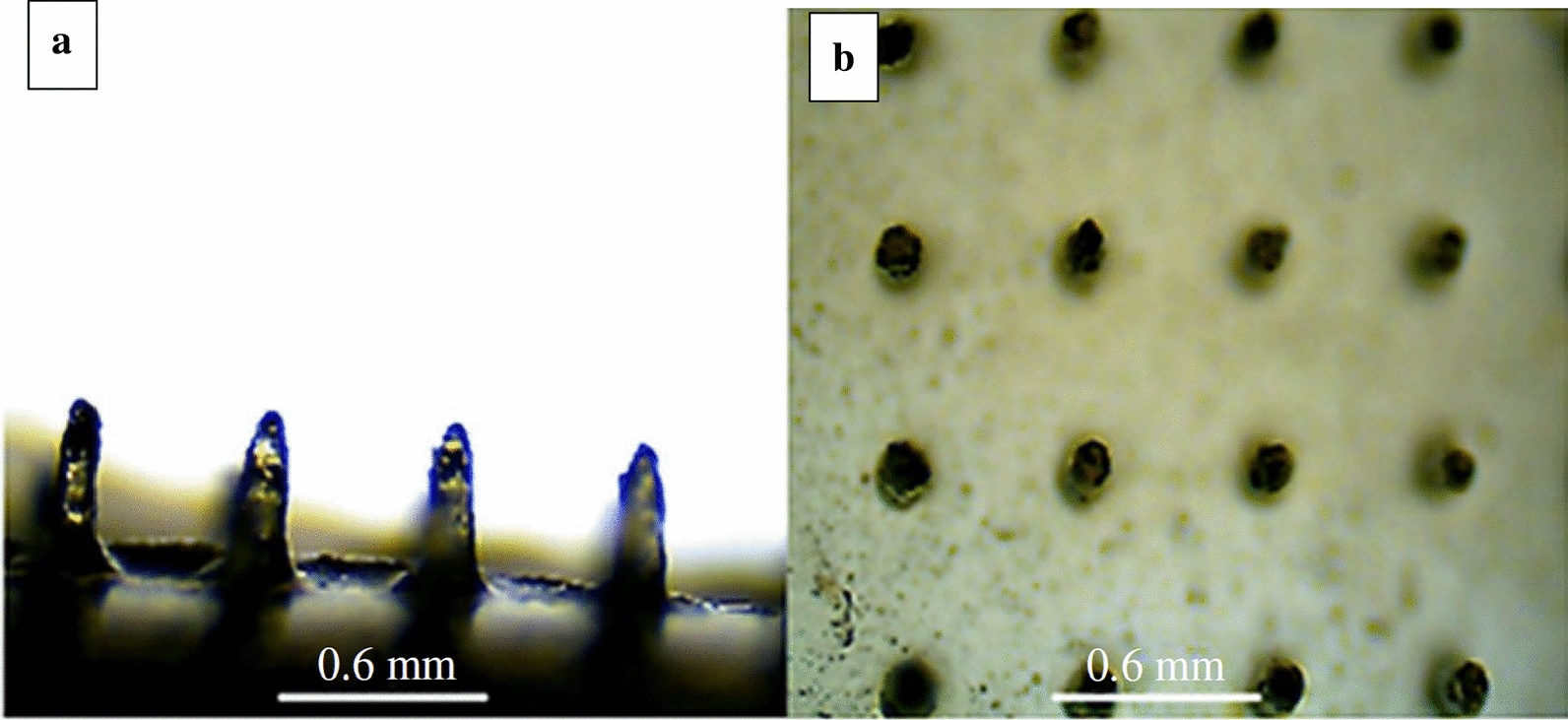
Optical micrographs of the CH-MGQDs microneedle array: **a** side view and **b** plan view (reprinted/reproduced with the permission of Ref. [201], copyright 2018, Interface Focus)

Based on MGQDs, Justin et al. [201] also combined chitosan (CH) and water-soluble PEG ring to synthesize a biodegradable detachable microneedle array, CH-MGQDs (Fig. 28). The release of CH-MGQDs to small molecular weight (MWt) drugs such as LA and bovine serum albumin (BSA) was also investigated. The results indicate that CH-MGQDs with intrinsic photoluminescent and supermagnetic properties are potential materials for the development of multifunctional microneedles for targeted and tracking percutaneous administration.

The advantages and disadvantages of different drug delivery-release modes are compared (Table 3). Given the low pH of the tumor microenvironment, researchers have developed an EPR-pH delivery-release mode. The drug loading efficiency in this mode is ~ 82.5% [41], and the drug release efficiency at pH 7.4 is 99% at 24 h in the previous report [184]. However, a small part of the drug will be released early due to pH changes during delivery. Considering that ligand-receptor binding enables more accurate drug delivery, a ligand-pH delivery-release mode was developed. Unfortunately, the drug loading efficiency and release efficiency decreased to 60% and 50% [193], respectively. Moreover, such DDRS can effectively release a large amount of antitumor drugs only after the low pH microenvironment of the tumor is formed. With the development of light-to-heat conversion materials and magnetic-to-heat conversion materials, EPR-Photothermal delivery-release mode have emerged. Although it cannot target antitumor, it avoids the disadvantages of relying solely on pH to release the drug, which can improve drug release and thus improve the efficiency of chemotherapy. The drug loading efficiency and release efficiency were 70.8% and ~ 40% (3 h), respectively [123]. The previously reported drug loading efficiency reached ~ 97% [196]. The core/shell-photothermal/magnetic thermal delivery-release mode exhibits excellent anti-tumor potential. Not only does it avoid the premature release of the drug before it is delivered to the tumor site, it also enables drug release and tumor cell thermal ablation at specific sites. For other diseases, other methods have also been developed, such as oral preparations and microneedle array. In terms of current reports, both drug loading efficiency and release efficiency still need to be greatly improved. In addition, the impact of the microenvironment in animals on DDRS is still unclear, and DDRS research needs to be transferred from the cell research stage to the animal research stage.

**Table 3:** Advantages and disadvantages of different drug delivery-release modes

Delivery-release mode	Load core	Load mode	Delivery mode	Release mode	Load/Release efficiency	Advantages	Disadvantages
EPR-pH	GQD	pH	EPR	Low-pH	Load efficiency: ~ 82.5% [41] Release efficiency: 60% (pH 7.4, 9 h), 100% (pH 7.4, 48 h) [184]	The most widely used drug delivery-release mode	The drug is sometimes lost during the delivery, which leads to a decrease in the treatment efficiency
Ligand-pH	GQD	pH	Ligand-receptor	Low-pH	Drug loading value: 16.6 wt% [191] Drug loading value: 21 wt% [192] Drug loading efficiency: 64%, Release efficiency: 50% (pH 7.4, 48 h) [193]	It can accurately deliver drugs	The drug is sometimes lost during the delivery, which leads to a decrease in the treatment efficiency
EPR-Photothermal	GQD	pH, Hydrophobic interaction	EPR	Photothermal	Drug loading efficiency: 70.8%, Release efficiency: ~ 40% (pH 7.4, 3 h) [123] Drug loading efficiency: ~ 97% [196]	It can control the release of drugs through NIR radiation, which reduces the early release of drugs and improves the efficiency of drug treatment	Because of lacking ligand and does not have a targeting function
Core/shell-photothermal/Magnetic Thermal	Core/shell structure	Stirring	Magnetic field	Photothermal/magnetic Thermal	Drug loading capacity: 47 μg mg^−1^ [141]	It can effectively prevent the early release of drug during the delivery, and could enhanced the tumor treatment efficacy through significantly improved the cell penetration efficiency and cell absorption capacity	The load core is relatively new, and animal testing is not enough

In addition, GQDs have PL characteristics; therefore they can track drugs for biological imaging. GQDs have limited functions, hence, in order to design multifunctional DDRS, multiple elements and groups are usually doped inside the GQDs. Moreover, even though the simulation results can provide good insights about the possible treatment efficacy in vitro*,* the practical in vivo approaches should not be overlooked. At present, the performance of some potential DDRSs are successfully confirmed through in vitro tests, however, due to the more complexity of in vivo tests the performance of testing DDRS in tumor-bearing mice needs to be more accurately studied.

### Gene, peptide, and other drugs delivery

Although most researches focus on tumor-targeted drug delivery, GQDs have shown promising potentials for delivery of other molecules such as DNA, peptides. An important technique of gene therapy is to delivery nucleic acids (DNA or RNA) to cells to restore or add gene expression, for the purpose of treating disease. Owing to its small size, GQDs possess the ability to cross the blood–brain barrier (BBB), this allows to ensure delivery of the nucleic acid payloads into cell cytosols and nuclei. Together with its low toxicity, good stability and luminescence properties which enable easy monitoring of drug release, GQDs is an excellent candidate for gene carriers. Plasmid DNA (pDNA) was proved to be conjugated with GQDs and MPG-2H1 chimeric peptide to form the GQDs-peptide-pDNA complex via non-covalent interactions. GQDs were found to allow efficient tracking due to their green and red emissions and enhance internalization of the plasmid harboring firefly luciferase gene into HEK 293T cells. This study suggested the GQDs-peptide-pDNA complex could be a promising vector for enhanced the transfection efficiency and tracking of pDNA in vitro and in vivo [126].Glycine–proline–glutamate (Gly–Pro–Glu, GPE), as a neuroprotective peptide, was conjugated with GQDs in a recent study. The results indicated that GQDs could be used as a novel nanocarrier to delivery neuroprotective peptide GPE to Central Nervous System (CNS), the GPE-GQDs can inhibit the aggregation of amyloid-β (Aβ) and reduce the inflammatory response, further protect the synapse and promote the neurogenesis [202].

GQDs also showed promising potential in transport Vanadium compounds, a kind of anti-diabetic agents. Vanadium coordination compounds [VO(p-dmada)] were proved to be packed on one side of the GQD sheets by the π–π stacking interaction. In vivo tests suggested that GQD-VO(p-dmada) exhibited improved pharmaceutical properties and enhanced anti-diabetic effects compared to VO(p-dmada) alone [203].

At present, the research and application of GQDs in drug delivery is still in its initial stage, however, the great potential of GQDs based nanocarriers has been proved already, we have reason to believe that more types of drugs can be delivered by this novel nanomaterial.

## Conclusion and prospect

This review focuses on the recent advances in the synthesis of GQDs and their applications in drug delivery. Several fabrication methods of GQDs are introduced, while some popular techniques such as hydrothermal method, microwave method, and molecular carbonization method are emphasized. Each of these fabrication methods has advantages and drawbacks that we should balance with the needs for a specific application. Furthermore, novel functionalization methods have been developed to improve the physicochemical characteristics of GQDs so that they can meet the high requirements for biomedical applications.

In addition, various drug release-delivery modes of GQDs-based drug release systems are reviewed. By analyzing the advantages and disadvantages of various types of DDRS, the trend of designing new multifunctional DDRS is pointed out. GQDs have been proved to be able to not only delivery anticancer drugs in various DDRS modes, but also act as nanocarriers to transport gene, peptides, and other non-anticancer drugs. However, before their practical applications in biomedicine and clinical practice, many challenges need to be addressed. Despite of the large improvement in the biocompatibility of GQDs, systematic studies on their potential long-term toxicity and how GQDs affects the immune systems, reproductive systems, and nerve systems in different animal models are needed. GQDs synthesized with different ways exhibited tremendous variations in their physic-chemical properties. Conflicting claims with respect to the biological properties of GQDs have been reported, such as their inherent antibacterial ability. Therefore, a standard for characterization of GQDs is need and would help to understand more clearly about the various GQDs researches. In addition, the size of GQD has great influence on its toxicity, surface functionalization and the ability to across biological barriers. More systematic studies involving the size of GQDs are still need in the future.

Although, DDRS research for GQDs is still at its infancy, it is speculated that the future research can successfully resolve the current problems faced by implementation of GQDs to design more safe and simple synthesis routs for efficient and mass-production of DDRS.

## Data Availability

The datasets used and/or analysed during the current study are available from the corresponding author on reasonable request.
